# Quantitatively controlled and measured-traumatic brain injury impairs adult neurogenesis and alters neuropathological signatures in mice

**DOI:** 10.7150/thno.114693

**Published:** 2026-01-01

**Authors:** Sungwook Yang, Suhyun Kim, Uiyeol Park, Hyeonjoo Im, Hyesun Cho, Kyung Eun Lee, Junsang Yoo, Seung Jae Hyeon, Se Jeong Lee, Im Joo Rhyu, Junghee Lee, Ann C. McKee, Eui-Sung Yoon, Hoon Ryu

**Affiliations:** 1 Center for Humanoid Research, Artificial Intelligence and Robot Institute, Korea Institute of Science and Technology (KIST), Seoul 02792, Republic of Korea.; 2 Laboratoy for Brain Gene Regulation and Epigenetics, Center for Brain Disorders, Brain Science Institute, KIST, Seoul 02792, Republic of Korea.; 3 Department of Anatomy, College of Medicine, Korea University, Seoul 20841, Republic of Korea.; 4 Advanced Analysis Data Center, KIST, Seoul 02792, Republic of Korea.; 5 Boston University Alzheimer's Disease Research Center and Department of Neurology, Boston University Chobanian & Avedisian School of Medicine, Boston, MA 02118, USA.; 6 VA Boston Healthcare System, Boston, MA 02130, USA.; 7 Center for Brain Convergence Research, Brain Science Institute, KIST, Seoul 02792, Republic of Korea.; 8 KHU-KIST Department of Converging Science and Technology, Kyung Hee University, Seoul 02447, Republic of Korea.; 9 Department of Medicine, Hanyang University Medical School, Seoul 04763, Republic of Korea.

**Keywords:** traumatic brain injury (TBI), closed-head injury model, adult neurogenesis, phosphorylated Tau (p-Tau), axonal damage

## Abstract

**Rationale:** Traumatic brain injury (TBI) poses a significant global health concern, necessitating a comprehensive understanding of its pathophysiology to devise effective treatments. The correlation between the intensity of head impact during injury and resultant neuropathology and behavioral changes in TBI remains unclear.

**Methods:** The Quantitatively Controlled and Measured-TBI (QCM-TBI) system, a novel closed-head injury model, enables precise control and measurement of impact during collision. The QCM-TBI system is designed with a unique gravity-compensating animal support system that replicates natural head motion in human TBI. Using QCM-TBI in conjunction with a multimodal sensor fusion technique, we measured instantaneous force over the time of collision, while compensating distortion led by extreme acceleration of the force sensor. To address whether TBI affects neuropathology and behaviors of mice in a force-dependent manner, we conducted transcriptome analysis, electron microscopy, and confocal microscopy in QCM-TBI model. We further compared molecular and pathological features of QCM-TBI mice with chronic traumatic encephalopathy (CTE) patients.

**Results:** Transcriptome analysis of QCM-TBI mice showed a significant downregulation of neuronal genes associated with synaptic function and neurogenesis, particularly in the hippocampus, which correlated with the severity of neuropathological features. Molecular and neuropathological characteristics of QCM-TBI mice partially resemble those of chronic traumatic encephalopathy (CTE) patients. Levels of phosphorylated Tau (p-Tau) and amyloid precursor protein (APP) correlate with impact magnitude, while neurofilament levels diminish in QCM-TBI mice. Neurons exhibit ultrastructural axonal damage in an impact-dependent manner.

**Conclusions:** Overall, this study suggests head impact intensity leads to decreased adult hippocampal neurogenesis, increased levels of phosphorylated Tau (p-Tau), and axonal damage, reflecting key neuropathological signatures of traumatic brain injury. Consequently, therapeutic strategies for TBI should account for the impact's severity in determining neuropathological outcomes.

## Introduction

More than 50 million cases of TBI are reported over the world annually 1. Single or repetitive TBI can lead to both pathological and psychological changes, although the precise mechanisms have not yet been fully understood. For instance, repetitive TBIs in humans may result in CTE, a progressive neurodegenerative disease characterized by symptoms like depression, memory loss, parkinsonism, as well as gait and mood disability 2-6. Notably, this disease is particularly common among professional athletes involved in contact sports and military veterans 7-11. Furthermore, both single and repetitive TBIs have been identified as risk factors for Alzheimer's disease (AD) and other neurodegenerative disorders 12, 13. The postmortem examination of CTE patients' brains has revealed similar neuropathological features to those seen in AD, including elevated levels of p-Tau and diffuse senile plaques 12-16.

To better understand the relationship between impact and neuropathological and behavioral sequelae of concussion and head injury in humans, animal models have been established over the last few decades 17-24. However, none of these models currently provide a quantitative measure of the actual impact, which is necessary to establish a causal correlation and develop potential therapeutics for TBI 20-22. Instead, most TBI animal models rely on applied velocity to estimate the degree of impact, despite the fact that the amount of impact can vary significantly based on the species, weight, and age of the animal, as well as the characteristics of the supporting structures used during impact 25, 26. While a few TBI animal models do use acceleration as a measure of impact, this measurement alone does not provide a complete understanding of the net impact delivered to the animal's head or how the applied force changes over the duration of the collision 22, 26, 27. Therefore, new animal models are needed that can accurately measure and quantify the impact of head injury, including net impact and changes in applied force over time.

One of the major challenges in TBI research is determining whether pathophysiological and behavioral changes occur in an impact-dependent manner 22, 25, 26. This is a crucial question from a clinical perspective, as patients with TBI exhibit a wide range of neuropathological and behavioral phenotypes, yet we lack precise information on how the magnitude of head impact or force at the time of injury is correlated with the resulting pathology and behavior. The mechanical impacts of TBI are directly associated with neuronal or diffuse axonal damage and vascular disruption 26, 28-30, which can result in long-term cognitive and motor impairments. Moreover, after the initial impacts of TBI, the regional environment of the brain undergoes changes mediated by various cell types such as neurons, astrocytes, microglia, oligodendrocytes, and the vascular unit 31-33. However, it is not yet fully understood how these cell types are pathologically affected by TBI in an impact-dependent manner 20, 22, 25, 26, 34. To elucidate the relationship between impact and TBI pathophysiology, further research is needed to investigate the impact-dependent changes in brain cell types and their contribution to the resulting pathology and behavior.

In the current study we have developed a novel engineered TBI system, the Quantitatively Controlled and Measured-Traumatic Brain Injury (QCM-TBI), which overcomes the lack of controllability and limited awareness of mechanics in previous TBI models. Our system accurately measures the impact of TBI under natural head concussion *in vivo*. To identify the molecular properties and propose biological markers of QCM-TBI, we conducted whole RNA sequencing analysis of the hippocampus in the QCM-TBI model. Additionally, we performed a comparison analysis to identify common transcriptome signatures between QCM-TBI and CTE using whole RNA sequencing data from post-mortem human brain temporal cortex tissues. We also investigated the relationship between changes in neurogenesis- and synapse-related gene signatures versus different dosages of mechanical force in the QCM-TBI animal model. Our study revealed a novel finding that both immature and mature neurons are negatively affected by TBI in a force-dependent manner, while other non-neuronal cell types, including astrocytes and microglia, are morphologically activated.

## Materials and Methods

### Design and principle of the QCM-TBI system

**Figure 1:**
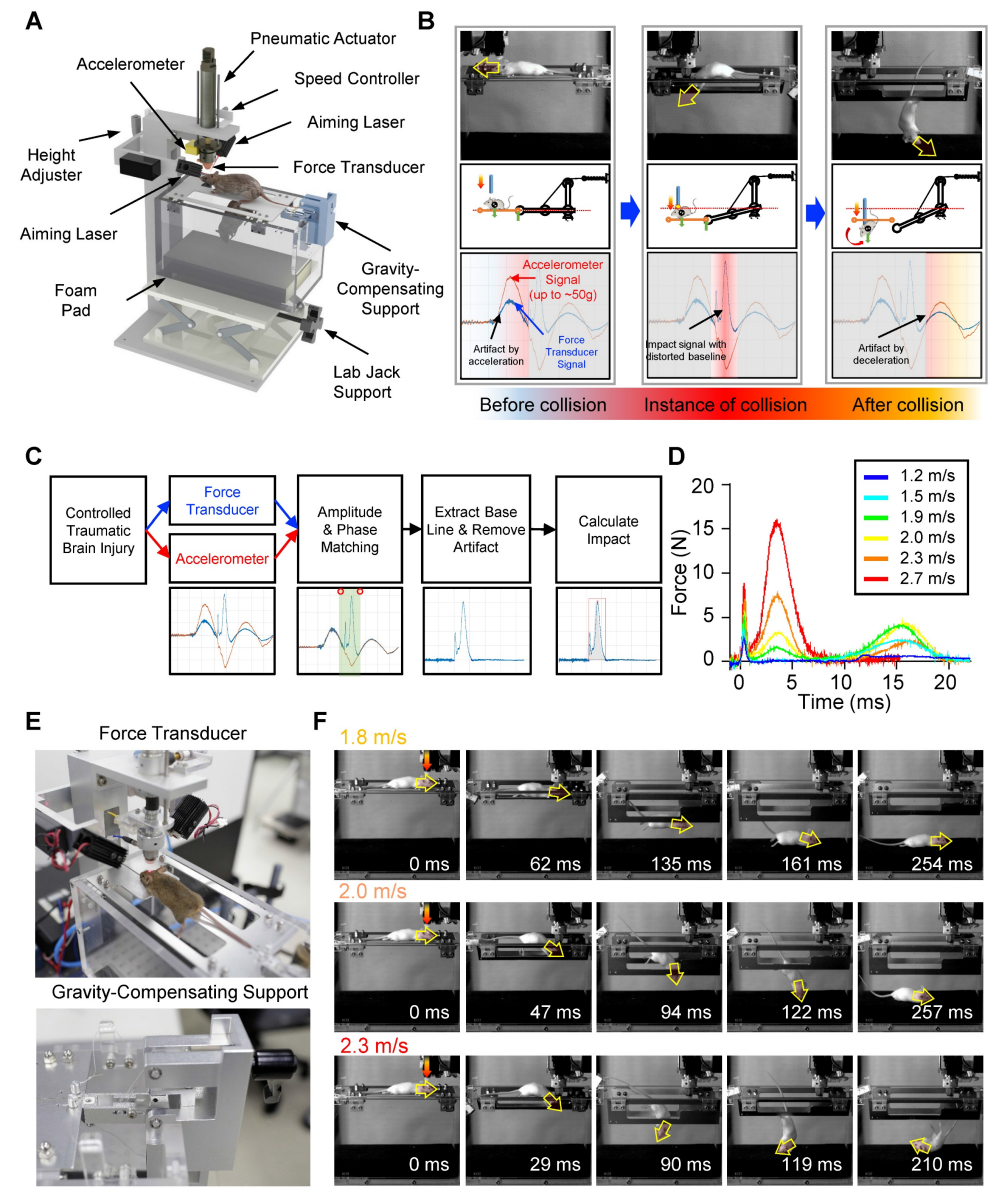
Quantitatively controlled and measured traumatic brain injury (QCM-TBI) with a gravity-compensating support mechanism.** A** Quantitatively controlled and measured traumatic brain injury system (QCM-TBI) with the 3D model of the QCM-TBI system. **B** Principle of the gravity-compensating animal support. The animal was initially laid on a pair of supporting plates, which were opened on each side by the vertical advancement of the impactor. A gravity-compensation mechanism balanced the weight of the animal without any external force until the animal fell and rolled over through the gap created by the opening plates. **C** Flow chart to measure injury impact loaded by the highly accelerating pneumatic actuator with the extra accelerometer. The optimization algorithm is applied to compensate for the distorted baseline of the impact sensor by high acceleration. **D** Impact profiles measured by QCM-TBI for various applying velocities. **E** Experimental setting with the mouse. A pair of laser modules were used to precisely target the spot of impact and to maintain the consistent distance between the impactor and the targeted spot (*upper*). Custom-built gravity-compensation mechanism for the TBI animal support (*lower*). **F** Still images of high-speed camera recording during impact by QCM-TBI. Translational acceleration was prominent with the low applied velocity-1.8 m/s. Rotational acceleration became noticeable as increasing applied velocity.

To deliver a reproducible and quantitatively controlled closed-head traumatic brain injury (TBI) in mice, we developed a custom-engineered QCM-TBI apparatus composed of three primary subsystems: (1) a controlled TBI-inducing system, (2) a measurement system for quantitative measurement of the impact, and (3) a gravity-compensating animal support for allowing the natural motion of the animal at the instant of impact (Figure 1A, Figure S1A-B). The TBI-inducing system applies controlled closed-head injury to the animal model. It is equipped primarily with a pneumatic actuator (DSN-20-50-P, Festo AG & CO. KG, Germany), a speed controller, an accelerometer (ADXL377, Analog Device, Inc., USA), and a pair of laser modules (Figure 1A). The pneumatic actuator expels an impactor to strike the head of the animal with high-speed impact. The speed controller attached to the actuator is used to adjust the velocity of the impactor, which results in a controlled impact on the animal head. To accurately aim a targeted spot on the head of the animal, the pair of laser modules are arranged at each side of the actuator. The laser spots of the modules are set to be coincident with each other at a specific distance between the impactor and the head of the animal. This system allowed reproducible positioning of the impactor in both single and repetitive TBI experiments, minimizing variability due to skull asymmetry or angle of contact. A schematic illustration of this setup is provided in Figure S1, and its implementation was critical for maintaining consistent impact dynamics and enabling reliable comparison across animals and conditions.

The measurement system consists of a high-sensitivity force transducer (ICP^®^ Force Sensor 208C02, PCB Piezotronics, Inc., USA) mounted at the distal end of the impactor. To deliver impact evenly onto the sensor, a hemispherical plastic tip is attached to its surface. During operation, the impactor rapidly accelerates to reach up to 2.7 m/s over a void distance of 10 mm, generating inertial acceleration of approximately 50 g (~500 m/s²). This extreme acceleration introduces significant artifacts in the raw force signals, even in the absence of collision (Figure 1B). To compensate for this, we implemented a sensor fusion approach by integrating the transducer with the mid-mounted accelerometer on the impactor. An optimization algorithm aligns the magnitude and phase of both sensor signals, allowing the accelerometer data to correct the distorted baseline of the force transducer (Figure 1C). The resulting artifact-free force profile enables accurate computation of the net impulse—the total impact energy—by numerical integration over the collision duration (Figure 1D).

The gravity-compensating animal support provides minimally constrained, gravity-neutral suspension of the animal body (Figure 1B). This mechanism enables natural head movement at the moment of impact by eliminating rigid fixation, ensuring physiologically relevant injury dynamics (Figure 1F). This setup ensures physiologically relevant injury dynamics while avoiding the rigid constraints common in other TBI models. Impact triggering is controlled via a PC interface and data acquisition board (NI USB-6361, National Instruments Corp., USA), which activates a solenoid valve to initiate the pneumatic strike. Real-time velocity monitoring is enabled through the embedded accelerometer.

### Sensor fusion with nonlinear optimization

To address the signal distortion caused by high inertial acceleration, we developed a nonlinear optimization-based sensor fusion algorithm. An optimization technique was adopted to match the different modalities of the two sensor signals—the magnitude and phase of the accelerometer signal with those of the force transducer. The matched profile of the accelerometer was then used to correct the fluctuating baseline of the highly accelerating force transducer. To achieve this alignment, we defined an objective function 
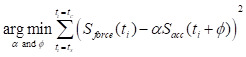
to minimize the squared error between the force transducer signal and the amplitude- and phase-adjusted accelerometer signal, where 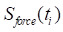
and 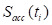
are the signals of the force transducer and the accelerometer, respectively, at the time 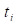. In this formulation, 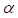
represents the amplitude scaling factor and 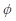
denotes the phase difference between the two signals. The optimization was confined to a specific window—ranging from the onset of acceleration to just before the moment of collision—to exclude mismatched signals during physical contact. The start of acceleration 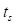
was identified by detecting the earliest significant rise above baseline in the force signal while scanning backward from the first peak. The start of collision 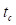
was determined as the first major peak within the range bounded by the prior acceleration-induced peak and subsequent deceleration valley. These two time points defined the fitting interval for optimization. The optimal amplitude and phase parameters were estimated by performing unconstrained multivariable optimization using the '*fminserch.m*' function in MATLAB^®^. Once the optimal parameters were obtained, the corrected force signal with a stabilized baseline was reconstructed as 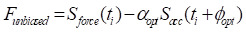. Finally, the net impulse 
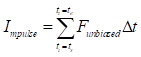
was then computed by numerically integrating the corrected force 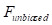
over the duration of collision using a sampling interval Δ*T* of 2 ms, where 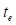
denotes the end of collision.

### Gravity-compensating animal support

To ensure high reproducibility and accuracy in our experiments, we designed the QCM-TBI apparatus with a gravity-compensating animal support that replicates cervical flexion/extension during impact. The animal was initially placed on a pair of supporting plates, and the weight of the animal was balanced by a gravity-compensation mechanism, which remained in place until the plates were opened (Figure 1E, Figure S1A- B). This allowed the animal to fall and roll over through the gap created by the opening plates (Figure 1F). By opening the supporting plates on each side through the vertical advancement of the impactor, we were able to replicate the natural body motion of the animal with cervical flexion/extension, as typically observed in human head collision (Figure 1F). This ensured that our experiments were highly accurate and reproducible.

### Animal

To ensure ethical standards were met, the animal study procedures were approved by Institutional Animal Care and Use Committee (IACUC) in the Korea Institute of Science and Technology (KIST) (Approval number: KIST-IACUC-2024-001). Eight-week-old male C57BL/6 mice were used in the study and were anesthetized with 2% Avertine (23 μg/g) via IP injection prior to QCM-TBI. The sham-injured group underwent the same anesthesia administration but received no impact. In the context of TBI, such hormonal influences may confound the interpretation of injury severity, inflammatory responses 8, and neurogenic recovery 11,12. Therefore, male mice were selected to provide a more stable baseline for force-dependent comparisons in the QCM-TBI model.

**Figure 2:**
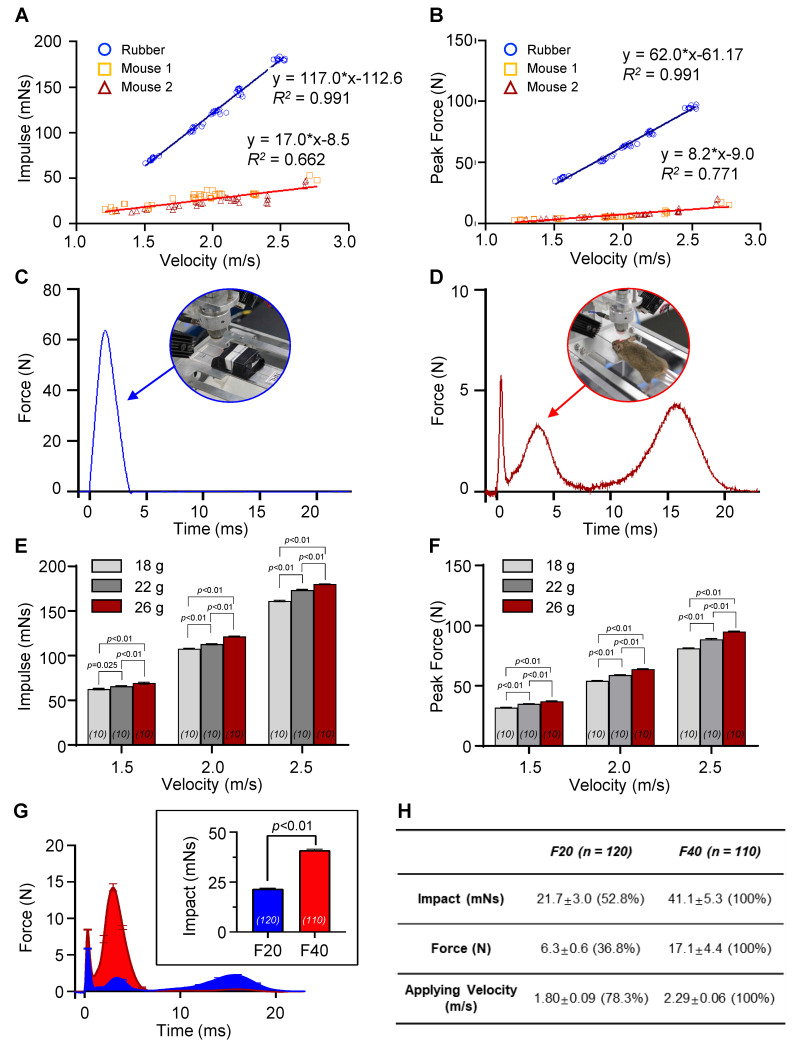
Model-dependent impact dynamics by QCM-TBI. **A, B** The net impact and the peak force were measured in two different types of experimental models—a rubber mock-up and two mice—varying applied velocities from 1.2 to 2.7 m/s. Morbidity was not observed with a single episode of impact with the maximum velocity of the apparatus ~ 2.7 m/s during the experiment. **C, D** The solid object made of rubber exhibited a single sharp peak during impact, whereas the animal model showed multiple force peaks due to repetitive head collisions by whiplash. These results indicated that rotational head motion by cervical flexion/extension in vertebrae significantly reduces the amount of impact and the peak force during impact. **E, F** The net impact and peak force were measured for the rubber mock-up models with the different weights (18, 22, and 26 g), while varying applying velocities from 1.5 to 2.5 m/s (*n* = 10). Both the net impact and peak force became higher as the applying velocity increased. The net impact and peak force of the three mock-up models with different weights significantly differed even for identical applied velocities **G** Average force-time (FT) profiles for the two sets of impulse for pathological study. The inset indicates the comparison of net impulse for two doses (21.7 mNs and 41.1 mN—represented by F20 (*n* = 120) and F40 (*n* = 110), respectively). **H** Quantitative analysis of two sets of TBI doses, F20 and F40. F20 resulted in a 50% net impact compared with F40. Data represent the mean ± SEM.

The QCM-TBI mouse model was developed according to experimental procedures and time schedules (Figure 4A), where mice were subjected to QCM-TBI five times with a three-day interval. To validate the delivery of graded mechanical force in our QCM-TBI model, we analyzed the real-time force-time (FT) profiles across experimental groups. The average force traces exhibited distinct peak amplitudes and durations between the two target impulse levels (Figure 2G). The net impulse, calculated by integrating the force over time, was 21.7 mNs for F20 (*n* = 120) and 41.1 mNs for F40 (*n* = 110), respectively. Quantitative comparison confirmed that F20 induced approximately 50% of the net mechanical impact delivered in the F40 group (Figure 2H), validating the dose-controlled nature of our QCM-TBI system.

To ensure reproducible targeting of the impact site across experimental trials, we implemented a laser-guided positioning system integrated into the QCM-TBI apparatus. Intersecting laser beams were aligned with the midline region of the dorsal skull, precisely between bregma and lambda, to maintain consistent spatial positioning of the impactor (Figure S2C). High-magnification images confirmed accurate laser alignment on the skull surface (Figure S2D), and localized skin contusion was not consistently observed at the targeted site following QCM-TBI (Figure S2E), further supporting the reliability of this targeting method.

### RNA isolation and library sequencing of QCM-TBI samples

Total RNA was extracted from the dorsal hippocampus of QCM-TBI mice using Trizol reagent (Invitrogen, USA) and its quality and quantity were determined using the Agilent 2100 bioanalyzer (Agilent Technologies, Amstelveen, The Netherlands) and ND-2000 Spectrophotometer (Thermo Inc., DE, USA), respectively. The NEBNext Ultra II Directional RNA-Seq Kit (New England Biolabs, UK) was used for library preparation, and the Poly(A) RNA Selection Kit (Lexogen, Austria) was utilized for mRNA isolation. The isolated mRNAs were subjected to cDNA synthesis and shearing according to the manufacturer's instructions, and the Illumina indexes 1-12 were used for indexing. The libraries were enriched using PCR and their mean fragment size was evaluated using the Agilent 2100 bioanalyzer (DNA High Sensitivity Kit, Agilent, USA). Library quantification was performed using the StepOne Real-Time PCR System (Life Technologies, USA), and paired-end 100 sequencing was conducted using the HiSeq X10 (Illumina, USA) for high-throughput sequencing.

### Differential gene expression analysis of QCM-TBI samples

We conducted RNA-Seq using dorsal hippocampal tissue samples from control (*n* = 2), F20 (21.7 mNs; *n* = 2), and F40 (41.1 mNs; *n* = 2) QCM-TBI mice, where F20 and F40 represent force conditions defined by net impulse as measured by the QCM-TBI system. Raw reads were mapped to the Gencode reference genome (Release M25, GRCm38) using STAR 35. Gene expression levels were estimated using HTSeq-count (Additional file 3)^36^. We used normalized read counts for the gene expression profiling using variance Stabilizing Transformation function of DESeq2 37. We considered differentially expressed genes (DEGs) as following criteria: 1) |Foldchange| ≥ 1.5 2) q-value < 0.05. Q-value is an adjusted p-value corrected for multiple testing using the Benjamini-Hochberg correction.

### Cell-type specific gene enrichment analysis

To identify cell type-specific genes, we employed a systematic classification approach that involved selecting the top 1000 genes for each of the five cell types: neuron, astrocyte, oligodendrocyte, microglia, and endothelial 38. The cell type-specific genes were identified through five RNA-seq data 39-43. We defined cell type-associated genes using three criteria: specificity, enrichment, and absolute expression. Specificity measures whether a gene is expressed exclusively in one cell type, while enrichment measures whether a gene has a higher expression level in one cell type than in all other types. Additionally, absolute expression measures whether a gene tends to have high expression in a particular cell type, regardless of its expression levels in other cell types.

### Differential gene expression analysis of CTE samples

The RNA sequencing data of CTE and normal post-mortem human brain samples were downloaded from the European Nucleotide Archive (ENA) accessions no. ERP110728. Sequencing reads were aligned to the human reference genome (GRCh37.p13) using the STAR alignment tool. PCR duplicates were removed using Picard Mark Duplicate, and filtered reads were processed for variant calling using GATK. For the gene expression profiling, we used normalized read counts by using the *varianceStabilizingTransformation* function of DESeq2 package. The normalized read counts were applied by principal component analysis using the most variable 500 genes. Supervised clustering was performed using dnet R package. DEGs were defined as gene with |Foldchange| ≥ 1.5, q-value < 0.05.

### Common genes data analysis of QCM-TBI and CTE

To identify common DEGs between QCM-TBI and CTE, we performed gene ontology (GO) pathway enrichment analysis using the Molecular Signatures Database 44. To visualize the results, we applied centered gene expression data to the average linkage clustering algorithm using Cluster 3.0 software 45, and the heatmap was created using Java Treeview 46. For the analysis, we calculated the Fragments Per Kilobase Million (FPKM) values for each gene using edgeR 47. The RNA sequencing data of QCM-TBI samples (controls, *n* = 2; F20, *n* = 2; F40, *n* = 2) are available under the European Nucleotide Archive (ENA) accessions no. ERP131298.

### Quantitative real-time PCR (qRT-PCR)

Total cellular RNA was isolated from cells using TRIzol reagent (Gene all, Korea). Fifty nanograms of RNA was used as a template for quantitative RT-PCR (qRT-PCR) amplification, using SYBR Green Real-time PCR Master Mix (Toyobo, QPK-201, Osaka, Japan). Primers were standardized in the linear range of the cycle before the onset of the plateau. Glyceraldehyde 3-phosphate dehydrogenase (GAPDH) was used as a control for cDNA loading and PCR. Real-time data acquisition was performed using a Light-Cycler 96 Real-Time PCR System (Roche Diagnostics, Indianapolis, IN, USA) (Applied Biosystems: 1 cycle at 50 °C for 2 min & 95° C for 10 min; 40 cycles at 95° C for 15 s & 60° C for 1 min). The relative gene expression was analyzed using the Light-Cycler 96 software and expressed as Ct, the number of cycles needed to generate a fluorescent signal above a predefined threshold.

### Immunohistochemistry (IHC)

Mice were deeply anesthetized and transcardially perfused with 4% buffered paraformaldehyde. Frozen brain tissues were sectioned in a coronal plane at 30 μm thickness. IHC was performed to examine neuronal and axonal pathology in mouse brain tissues. For chromogenic staining, endogenous peroxidase activity in the sections was inactivated by 3% H_2_O_2_ in TBS for 10 min. The tissue sections were blocked with blocking solution (5% BSA in TBST (0.3% Triton-X100 in TBS) for 1 hr and then further incubated with specific primary antibodies for p-Tau (S202/T205) (1:200, MN1020, Thermo Fisher Scientific, USA), p-Tau (S199) (1:500, ab81268, Abcam, USA), p-Tau (S396) (1:1000, ab109390, Abcam, USA), APP (1:500, NBP2-15575, Novus, USA), NEFM (1:500, ab64300, Abcam, USA), NEFL (1:1000, ab134460, Abcam, USA) and NEFH (1:200, NO142, Sigma Aldrich, USA), NEFL (1:1000, ab134460, Abcam, USA), NEFH (1:200, NO142, Sigma Aldrich, USA), and PDGFRαβ (1:100, ab32570, Abcam, USA), DCX (1:400, #4604, Cell signaling, USA), NeuroD1(1:200, #PAS-47381, Invitrogen, USA), neurofilament medium polypeptide (NEFM; 1:500, ab64300, Abcam, USA), synapsin1 (1:500, ab64581, Abcam, USA), Lamin B1 (1:500, Cat. No sc-374015, Sigma, USA), Fth1 (1:200, ab65080, Abcam, USA), IBA-1 (1:400, 016-26721, Wako, USA), GFAP (1:500, AB5541, Millipore, USA), OLIG2 (1:400, AB9610, Millipore, USA), MBP (1:200, AB980, Millipore, USA), and p-Tau (S202/T205) (1:200, MN1020, Thermo Fisher Scientific, USA) for 24 hr. Then, sections were incubated with HRP-conjugated secondary antibody for 1 hr for chromogenic staining or fluorescence-conjugated secondary antibodies included in Goat anti-Mouse IgG-Alexa Fluor® 488, Goat anti-Rat IgG-Alexa Fluor® 568 and Goat anti-Rabbit IgG- Alexa Fluor® 647 (1:500, Invitrogen, USA). After three washes, the tissues were processed with a Vector ABC Kit (Vector Lab, USA) for chromogenic staining. Immuno-reactive signals were detected with DAB chromogen (Thermo Fisher Scientific, USA). The image was observed under a bright field microscope (Olympus BX63, Japan). For BrdU assay, the brain sections were pretreated 2 N HCl for 1 hour at 37 °C and rinsed in 0.1 M borate buffer (pH 8.5) for 10 min before incubation in blocking solution. The sections were incubated with rat anti-BrdU antibody (1:400, Serotec, USA) with mouse anti-NeuN antibody (1:400, Millipore, USA) or rabbit anti -DCX (1: 200, ab153668, Abcam, USA) in blocking solution for 16 hours at 4 °C followed the blocking procedure.

### Chromogenic staining for post-mortem human brain tissues

Paraffin-embedded human postmortem brain tissues were sectioned in a coronal plane at 10 μm. 3% H_2_O_2_ in TBS was used to block endogenous alkaline phosphatase. Tissue sections were blocked with 2.5% normal horse serum (S-2000, Vector Laboratories, USA) for 1 h and then incubated with NeuN antibody (1:400, MAB377, Millipore, USA), PDGFRαβ (1:100, ab32570, Abcam, USA), p-Tau (S202/T205) (1:200, MN1020, Thermo Fisher Scientific, USA), p-Tau (S199) (1:500, ab81268, Abcam, USA), p-Tau (S396) (1:1000, ab109390, Abcam, USA), and APP (1:500, NBP2-15575, Novus, USA) for 24 h. After washing three times with TBS, tissue slides were processed with Vector ABC Kit (PK-4000, Vector Laboratories, USA). Immuno-reactive signals were developed with DAB chromogen (D7304, Thermo Fisher Scientific, USA). Stained tissue slides were gradually processed back to xylene through an increasing ethanol gradient [70%, 80%, 90%, 95%, and 100% (1 time)] and subsequently mounted. The chromogenic signals were examined under a light microscopy (BX63, Olympus, Japan) equipped with high definition (1920×1200 pixel) digital camera (DP74) (Olympus, Japan).

### Confocal microscopy

Immunofluorescence staining for DCX (1:400, #4604, Cell signaling, USA), NeuroD1(1:200, #PAS-47381, Invitrogen, USA), neurofilament medium polypeptide (NEFM; 1:500, ab64300, Abcam, USA), synapsin1 (1:500, ab64581, Abcam, USA), and lamin B (1:500, Cat. No sc-374015, Sigma, USA) was analyzed using a confocal microscope (Nikon A1R, Japan). Pre-absorption with excess target protein or omission of primary antibody was used to demonstrate antibody specificity and background generated from the detection assay.

### 3D-reconstrction analysis of neurite outgrowth

For neurite outgrowth analysis of doublecortin (DCX)-, glial fibrillary acidic protein (GFAP)- and allograft inflammatory factor 1 (IBA-1)-positive cells, z-series at 1μm intervals images were acquired with 40x lens of confocal microscopy system (FluoView1000, Olympus, Japan). All morphological analysis was performed by Simple Neurite Trace (SNT), a free software plugin of Fiji-ImageJ 48. More than 10 cells of DCX-positive cells and more than 3 cells of GFAP- and IBA-1-positive cells in DG of QCM-TBI and control mice (*n* = 5 per group) were analyzed for quantification of total neurite length, total branching points and number of intersections of neurites 49.

### Behavioral test

Mice were habituated for at least 1 hr in the experimental room with white noise before the behavioral test. The experimental apparatus was cleaned with an alcohol-water solution (70%) before placing each animal to avoid any odor clues from previous animals.

#### Latency to righting reflex (LRR)

To examine the latency of the righting reflex, we placed control and injured mice on their side in a cage. We recorded the moment of impact and measured the length of time until the mice rolled onto their abdomens.

#### Open field test (OFT)

The OFT apparatus, a white acrylic box (40 x 40 cm, 40 cm high walls), was virtually divided into nine equal squares by software (Ethovision version 13.0; Noldus Information Technology, Wageningen, The Netherlands) into two zones: the central (20 x 20 cm) and peripheral zones. Each mouse was placed individually in the center of the apparatus for observation. Each trial was recorded by video for 10 min to analyze the animal's total distance traveled (cm), velocity (cm/sec), and frequency (count) in the center zone.

#### Elevated plus maze (EPM)

The EPM apparatus comprised two equally sized open arms (35 x 5 cm) and two equally sized enclosed arms with 15 cm high walls. The height of the plus maze was positioned 55 cm above the floor. Each mouse was placed individually in the center of the equipment with the nose pointing at one open arm. Each trial was recorded by video for 10 min to measure the total distance traveled (cm), velocity (cm/sec), and time spent in the center zone, open and closed arms. Behavioral changes were analyzed by Ethovision software (Noldus Information Technology, USA).

#### Novel object recognition (NOR) & Nove object place recognition (NOPR) test

NOR and NOPR tasks were performed in a white open field box (40×40×40 cm) with slight modifications from the procedures. Two types of objects were different in shape, color and texture. One of them was a yellow regular tetrahedron, made of acryl. The other one was a black and red color sphere, made of urethane. The objects were fixed to the ground of the box, not to be moved by mice. Sniffing objects was considered as the explorative action of mouse. NOR test was composed of 3 steps such as habituation, training and test, and given once per day. During the habituation step, mouse was placed in an open field box for 10 min without objects. Then, during 2 times of training period, two identical objects were presented to the mouse for 10 min. At 2 h after the last training, one of the familiar objects was replaced with a novel object and presented to the mouse for 10 min for the test. Procedures for NOPR task were similar to NOR task except that one of the objects was moved to a different location for the test. The test was video-recorded with encoding software (Ethovision XT, Noldus, USA) and the results including total number of arm entries and alternation behavior were analyzed later.

### Transmission electron microscopy (TEM) analysis

Control and QCM-TBI-induced mouse brains were fixed in glutaraldehyde (2%) and paraformaldehyde (4%) followed by 1% OsO_4_. Ultrathin sections of Epon-embedded tissue were cut in a plane parallel to the surface of the slice to observe the extent of myelination and compact myelination. The sections were observed on a Jeol 100CX electron microscope (JEOL Ltd., Japan) at 80 kV. The axon diameter and the myelin sheath thickness were examined in at least 10 axons/slide/case. To calculate the G-ratio, a functional and structural index of optimal axonal myelination, the inner and total outer diameter of axons were measured in the same region of the spinal cord. And the number and area of outer and inner tongues of myelin were quantified in the same region 50.

### Statistical analysis

The data are presented as the mean 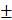
SEM. Data analysis was performed by Student *t*-test and one-way ANOVA followed by Fisher's protected least significant difference test using StatView 4 (Abacus Concepts, CA).

## Results

### Model-dependent impact dynamics

Although controlling the applied velocity is a crucial factor in regulating the impact, the characteristics of the objects in the collision, such as their structure, material, and weight, can cause significant variations in the resulting impact and force, even at identical applied velocities (Figure 2A-B and Figure S1C). To compare the impact and force between two different experimental models, a rubber mock-up and live mice, we measured the net impulse and peak force under varying applied velocities ranging from 1.2 to 2.7 m/s (Figure 2A-B). The rubber mock-up, which was made of solid rubber and represented a rigid structure, experienced much higher impact than the live mice, whose vertebrae facilitated cervical flexion/extension. Importantly, no morbidity was observed in the mice following a single episode of impact with the maximum velocity of the apparatus, 2.7 m/s. For instance, at an applied velocity of 2.0 m/s, the rubber mock-up displayed 3.4- and 11.0-times higher net impulse and peak force, respectively, compared to the mice. Moreover, the force profiles during collision were significantly distinct between the two models (Figure 2C-D). The rubber mock-up exhibited a single sharp peak during impact, whereas the animal model showed multiple force peaks due to repetitive head collisions caused by whiplash. These results suggest that rotational head motion resulting from cervical flexion/extension in vertebrae plays a significant role in reducing the amount of impact and the peak force during impact.

To investigate the impact of different object characteristics on the resulting force and impulse, we conducted experiments using various rubber mock-up models with different weights and live mice as the experimental models. We applied velocities ranging from 1.2 to 2.7 m/s to both models and compared the resulting net impulse and peak force. The results showed that, despite identical applied velocities, the amount of impact and force significantly varied due to the object's characteristics, such as structure, material, and weight (Figure 2A-B, Figure S1C). To further investigate the impact of object weight on the resulting force and impulse, we conducted experiments using various rubber mock-up models with different weights (18, 22, and 26 g) while varying the applied velocities from 1.5 to 2.5 m/s. We observed that both the net impulse and peak force increased with increasing applied velocity for all rubber mock-up models. However, we also found that the net impulse and peak force significantly differed among the three mock-up models with different weights even for identically applied velocities (Figure 2E-F). These results indicate that the characteristics of the object in the collision, including its weight, significantly affect the resulting force and impulse, and should be carefully considered when interpreting the experimental results.

### Velocity-dependent impact dynamics *in vivo*

The QCM-TBI model exhibited a correlation between applied velocity and the amount of impact delivered *in vivo* (Figure 2A-B). To further study the neuropathological and behavioral changes induced by QCM-TBI, we defined two levels of quantitative impact to be applied to the mouse brain, 21.7 mNs and 41.1 mNs, which resulted from applied velocities of 1.8 m/s and 2.3 m/s, respectively (Figure S1C). The peak forces of the impact were measured to be 6.3 N and 17.1 N on average for different degrees of impact (Figure 2A-B). The transient force profiles over the time of impact (force-time (FT) curve) considerably varied with applied velocities. For instance, the peak force was observed in the first collision at the lower speed, whereas the peak force in the secondary collision was observed at the higher speed (Figure 2C-D). Specifically, for the lower applied velocity, the FT curve revealed multiple collisions of the mouse head, while fewer collisions occurred for a short period of time for the higher velocity (Figure S1C). These could be explained by the instantaneous head motion of the animal during impact (Figure 2D). As the impactor was accelerating toward the head of the animal, the mouse head experienced more repetitive head collisions at the lower applied velocity due to less head rotation. Alternatively, the mouse head experienced a greater degree of angular rotation at the higher applied velocity, which yielded fewer multiple collisions but higher peak force and net impact for a short duration of collision (Figure 2C-D). To investigate force-dependent molecular responses, we compared transcriptomic profiles from mice subjected to two levels of controlled impact: F20 (21.7 mNs) and F40 (41.1 mNs), as measured by net impulse using our QCM-TBI system (Figure 2G-H). This classification allowed us to stratify animals based on biomechanical severity and assess dose-responsive gene expression changes.

### Identification of force-dependent genes and common gene signatures in QCM-TBI and CTE

**Figure 3:**
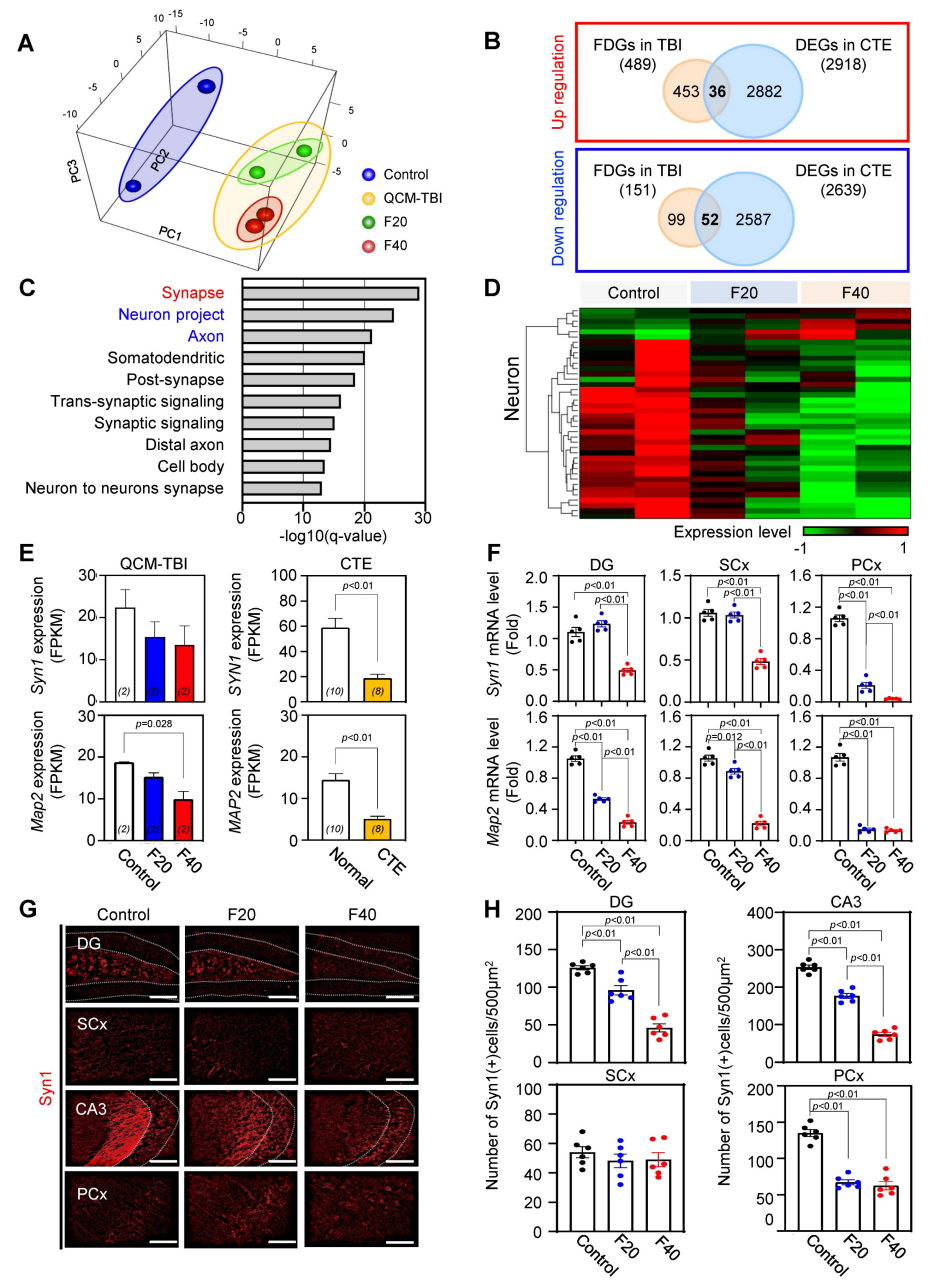
Synapse- and axon-associated gene profiles are significantly modulated by QCM-TBI. **A** Principal component analysis (PCA) exhibits apparent changes of transcriptome signatures by QCM-TBI. Control samples were shown in a blue circle and QCM-TBI mouse samples were in a yellow circle (green, F20 samples; red, F40 samples). **B** Venn diagram indicating the number of overlapped genes between force-dependent genes in QCM-TBI mice and differentially expressed genes in CTE patients. **C** GO enrichment analysis identified 50 commonly down-regulated genes in QCM-TBI mice and CTE patients. **D** Heatmap represents that 640 genes were altered in a force-dependent manner with |slope| > 1 and coefficient of determination (R^2^ > 0.8). **E** The FPKM levels of Synapsin 1 (*Syn1*) and *Map2* were decreased in QCM-TBI mice and CTE patients. **F** Quantitative RT-PCR analysis verified reduction of *Syn1* and *Map2* expression levels in the DG, SCx, and PCx of the QCM-TBI mouse model (control, *n* = 5; F20, *n* = 5; F40, *n* = 5). **G** Syn1 immunoreactivity was reduced in F20 and F40 QCM-TBI compared to control. White dot lines indicate granule cell layer in DG and pyramidal cell layer in CA3. Scale bars: 100 µm. **H** The number of Syn1-immunopositive neuronal cells was reduced in a force-dependent manner in the hippocampus of QCM-TBI mice. Data represent the mean ± SEM.

To identify force-dependent genes (FDGs) signatures of QCM-TBI, the researchers defined 640 FDGs based on |slope| > 0.2 and coefficient of determination (R^2^) > 0.8 (Figure 3A-C). Out of these, 489 FDGs were increased, and 151 FDGs were decreased depending on the force of QCM-TBI (Figure 3B). To verify whether the QCM-TBI mouse model is an appropriate TBI model for exhibiting molecular and neuropathological features similar to human CTE, we compared mouse RNA sequencing data with RNA sequencing data of 10 normal and 8 CTE post-mortem human brain tissue samples (Figure 3B and Figure S3A, C, D). To gain insight into the biological process in QCM-TBI and CTE, we observed that the expression patterns of CTE were distinct from normal, with 35% variance in the PC1 axis (Figure S3A, B) and found gene set enrichment analysis of 32 commonly *down*-regulated genes in both QCM-TBI and CTE (Figure S3C-D).

To identify responsive FDGs, we applied differential expression criteria of |fold change| ≥ 1.5 and q-value < 0.05, resulting in 38 upregulated and 60 downregulated DEGs between QCM-TBI (F20 and F40) and control groups (Figure S5A, Additional file 2 and 4). By comparing FDGs with DEGs (Figure S5A), we found 16 genes that were force-dependently up-regulated and 22 genes that were force-dependently down-regulated in QCM-TBI (Figure S5A). To validate the transcriptome results, we conducted immunostaining in the dorsal hippocampus of control and QCM-TBI mice (Figure S5B-D). We confirmed that the immunoreactivity of ferritin heavy chain 1 (Fth1) among several targets was markedly down-regulated in the hippocampal neurons of QCM-TBI mice (Figure S5B-D). The densitometry analysis showed that the Fth1 protein expression level was significantly decreased in a force dependent manner (Figure S5D).

To explore the common gene signatures of QCM-TBI and CTE, the researchers compared FDGs in QCM-TBI with DEGs in CTE (Figure 3B-D). They found 36 commonly up- and 52 down-regulated genes in QCM-TBI and CTE (Figure 3B). In addition, 2,918 up-regulated DEGs and 2,639 down-regulated DEGs were identified in CTE (Figure 3B). The GO enrichment analysis of 52 down-regulated genes in turquoise module according to QCM-TBI force and down-regulated DEGs in CTE was performed (Figure 3C). Among the top10 GO pathways, the synapse pathway was remarkably enriched in 31 down-regulated genes (Figure 3C-D). Notably, Syn1 and Map2 were significantly down-regulated genes in both QCM-TBI and CTE (Figure 3E). To validate our transcriptome results, we performed qRT-PCR validation of key force-dependent genes using independent cohorts with *n* = 5 mice per group. This validation confirmed consistent force-dependent regulation of *Syn1* and *Map2* (Figure 3F); *Dcx* and* NeuroD1*(Figure S5E-F), reinforcing the reliability of our transcriptomic findings with improved statistical support. We also examined the immunoreactivity of Syn1 in different brain regions and found a significant decrease in the dentate gyrus (DG), somatosensory cortex (SCx), cornu ammonis 3 (CA3) and the pyriform cortex (PCx) (Figure 3G-H). Specifically, Syn1 intensity decreased in a force-dependent manner in DG and CA3 compared to other regions (Figure 3G-H). These findings suggest that mechanical impact induces a more pronounced down-regulation of neuronal genes compared to non-neuronal cells.

### Effect of QCM-TBI on adult hippocampal neurogenesis

We performed Gene Ontology (GO) analysis on 32 neuron-specific genes that were downregulated in both QCM-TBI and CTE and found that these genes were associated with synaptic signaling, trans-synaptic signaling regulation, and neurogenesis pathways (Figure S5E). In this study, we focused on neurogenesis-related genes that were commonly altered in QCM-TBI and CTE (Figure S5E-F). To validate the transcriptomic findings, we examined mRNA expression of doublecortin (*DCX*) and neurogenic differentiation 1 (*NeuroD1*), key markers of immature neurons. Both *DCX* and *NeuroD1* mRNA levels were reduced in a force-dependent manner in the dorsal hippocampus of QCM-TBI mice (Figure S5F). Because adult hippocampal neurogenesis in the dentate gyrus is essential for learning and memory 51-53, we next assessed whether QCM-TBI affected neuronal and non-neuronal cell populations in this region. Analysis of cell-type-specific gene signatures showed that in neurons, 6 force-dependent genes (FDGs) were upregulated and 34 FDGs were downregulated following QCM-TBI (Figure 3D). Astrocytes, oligodendrocytes, and microglia also exhibited force-dependent transcriptional changes, with 22, 23, and 23 cell-type-specific genes altered, respectively (Figure S6C). Notably, 32 neuron-specific genes were commonly downregulated in both QCM-TBI and CTE, while only one was upregulated (Figure S3D). Gene set enrichment analysis of these 32 commonly downregulated neuronal genes revealed strong enrichment for pathways related to synaptic signaling, trans-synaptic regulation, and neurogenesis (Figure S5E, F).

**Figure 4:**
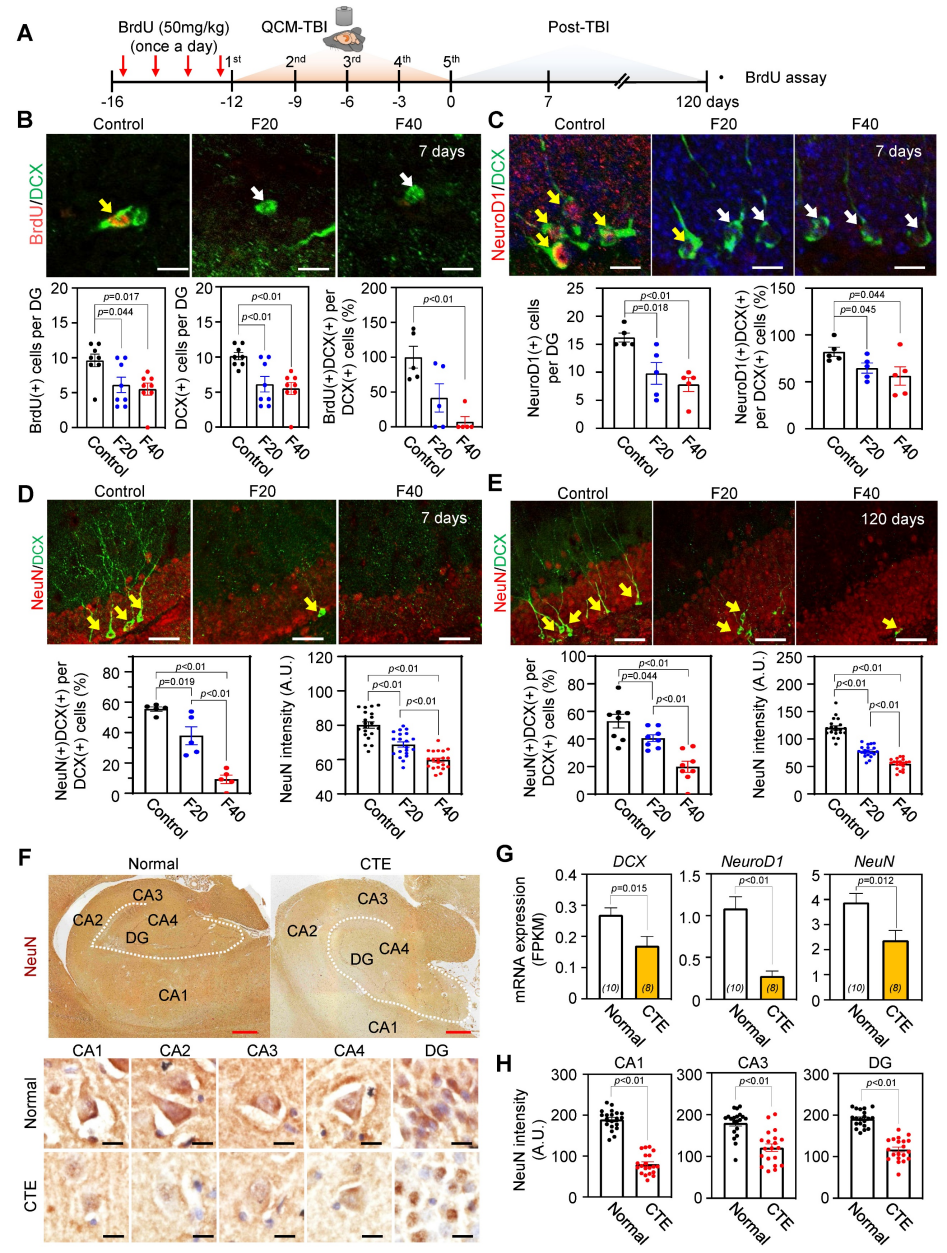
Adult neurogenesis-related genes are commonly deregulated in QCM-TBI mice and CTE patients.** A** Experimental scheme of BrdU injection and QCM-TBI for measuring of adult neurogenesis in hippocampus. **B** BrdU incorporation in DCX-positive neurons was significantly decreased by QCM-TBI (control, *n* = 5; F20, *n* = 5; F40, *n* = 5). Yellow arrows indicate double-positive cells, and white arrows indicate single-positive cells. Scale bars: 20 µm. **C** *NeuroD1* immunoreactivity in DCX-positive neurons was significantly decreased by QCM-TBI (control, *n* = 5; F20, *n* = 5; F40, *n* = 5). Yellow arrows indicate double-positive cells, and white arrows indicate single-positive cells. Scale bars: 20 µm. **D** *NeuN* immunoreactivity in DCX-positive neurons was significantly decreased by QCM-TBI (control, *n* = 5; F20, *n* = 5; F40, *n* = 5). Yellow arrows indicate double-positive cells. Scale bars: 50 µm. **E** QCM-TBI significantly reduced both NeuN and DCX immunoreactivity in the hippocampus (HPC) (control, *n* = 5; F20, *n* = 5; F40, *n* = 5). The long-term effect was determined 120 days after QCM-TBI. Yellow arrows indicate double-positive cells. Scale bars: 50 µm. **F** Representative image of NeuN immunostaining images in the hippocampus of CTE patients and normal. The lower panel shows a magnified image for each hippocampus region. White dot lines indicate outline of DG molecular layer. Scale bars: 2mm (*red*); 10 µm (*black*). **G** The FPKM levels of *DCX, NeuroD1* and* NeuN* were decreased in CTE compared with normal. **H** NeuN immunoreactivity was significantly decreased in the hippocampus of CTE patients (normal, *n* = 5; CTE, *n* = 5). Data represent the mean ± SEM.

We next investigated synapse- and neurogenesis-associated markers in the hippocampus after QCM-TBI (Figure 3-4). Immunostaining for neurogenesis markers (DCX, NeuroD1) and mature neuronal marker (NeuN), along with glial markers (GFAP; astrocytes), oligodendrocytes maker (OLIG2), and microglia (IBA-1), were performed (Figure 4, Figure S6A-D). To assess proliferation of immature neurons, BrdU was administered intraperitoneally (four daily injections) before the first QCM-TBI event (Figure 4A-B). The number of BrdU- and DCX-positive cells and the ratio of BrdU/DCX double-positive cells were significantly reduced in the dentate gyrus (DG) of QCM-TBI mice compared to controls (Figure 4B).

To evaluate neuronal differentiation, we examined NeuroD1 expressions in DCX-positive cells. Both the number of NeuroD1-positive cells and the proportion of DCX-positive cells co-expressing NeuroD1 were decreased in the DG of QCM-TBI mice relative to controls (Figure 4C). For neuronal maturation, we quantified DCX-positive cells co-labeled with NeuN and measured NeuN intensity at short-term (7 days post-TBI) and long-term (120 days post-TBI) (Figure 4D-E). Both the percentage of DCX/NeuN double-positive cells and NeuN intensity were reduced in QCM-TBI mice at both time points (Figure 4D-E). In contrast, no significant changes were observed in the number of IBA-1-, GFAP-, or OLIG2-positive cells in the DG, even at higher impact forces (Figure S6A-D). These findings indicate that neuronal cells, particularly those involved in neurogenesis and maturation, are more vulnerable to mechanical impact than non-neuronal cells in the QCM-TBI model.

### Neurogenesis-related gene expression decreased in brain tissues of CTE patients

To investigate whether neurogenesis-related genes are reduced in patients with CTE, we analyzed the FPKM levels of *DCX, NeuroD1*, and *NeuN* (Figure 4G) in brain tissues obtained from CTE patients and compared them to those of healthy controls. Our results revealed a significant decrease in the expression of these genes in CTE patients (Figure 4G). In addition, we observed a reduction in the immunoreactivity of NeuN in the hippocampus of CTE patients compared to healthy controls (Figure 4F-H). These findings suggest that the effects of neuronal cell damage observed in our QCM-TBI models are consistent with those observed in the hippocampus of CTE patients.

### QCM-TBI reduces neurite outgrowth and branching of DCX-positive cells

**Figure 5:**
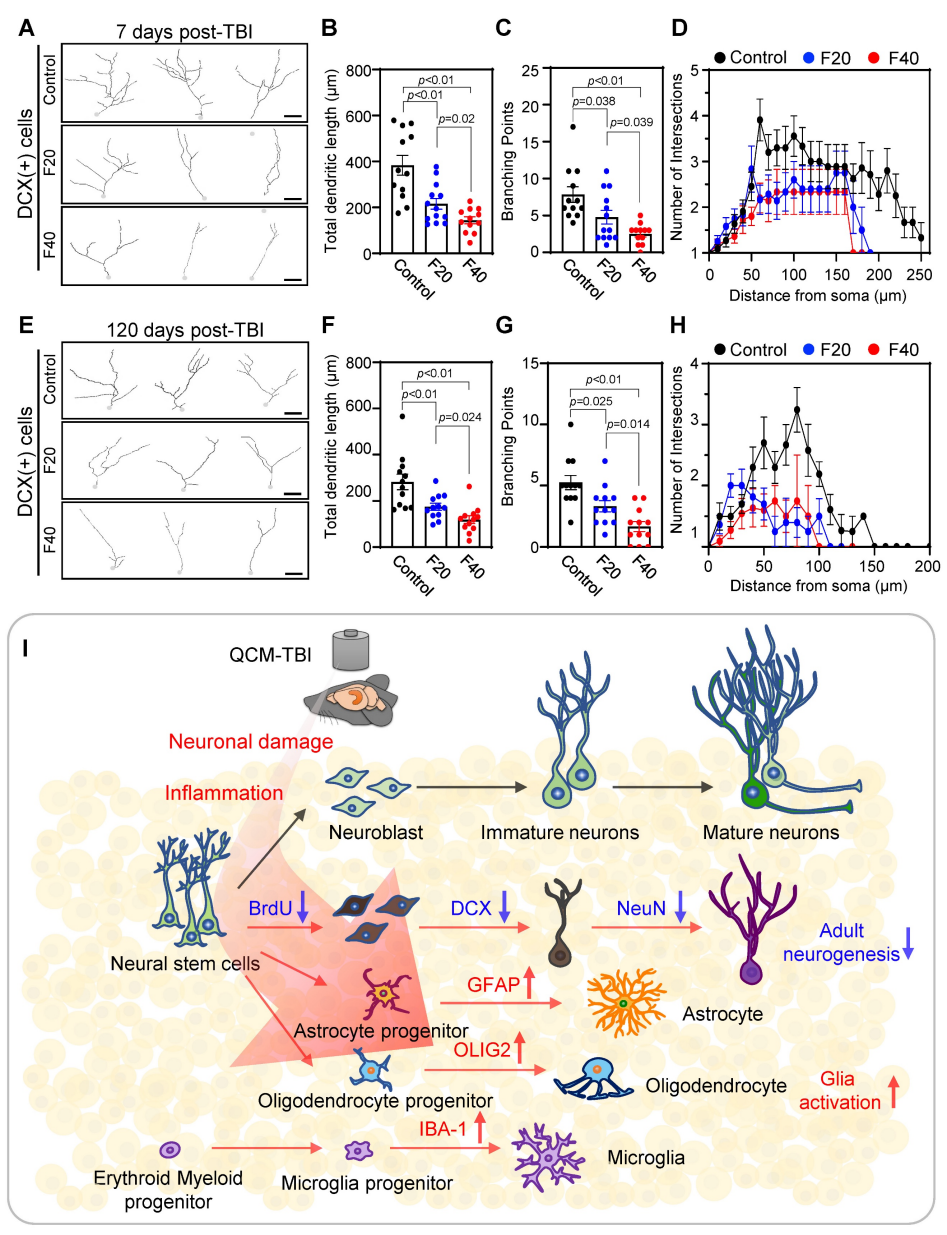
Neurite processes of DCX-positive cells are reduced in QCM-TBI mice.** A** 3D-reconstruction image of DCX-positive cells shows alteration of neurite processes by QCM-TBI. Scale bars: 20 µm. **B-D** The neurite length (B) and the number of branch points (C) and intersection (D) in DCX-positive neurons were reduced in a force-dependent manner by short-term effects of QCM-TBI. **E-H** The neurite processes of DCX-positive neurons were also reduced in a force-dependent manner by long-term effects of QCM-TBI. Scale bars: 20 µm. **I** Schematic representation of neurogenesis and associated molecular markers. In QCM-TBI mice, behavioral, biochemical, and pathological assessments revealed neuronal damage, including synaptic dysfunction and axonal injury. These damages are closely linked to changes in the hippocampal microenvironment affecting adult neurogenesis. To evaluate these effects, markers such as 5-bromo-2-deoxyuridine (BrdU), DCX, NeuroD1, and NeuN were used to identify neuronal cell fate, while GFAP, IBA-1, and OLIG2 were employed to assess glial cell populations, including astrocytes, microglia, and oligodendrocytes. Data represent the mean ± SEM.

To further investigate the effects of QCM-TBI on maturation of DCX-positive cells, we performed 3D-reconstruction analysis of neurite outgrowth using confocal images taken at 1μm z-steps in the DG of short-term and long-term QCM-TBI mice, as well as control mice (Figure 5A-H). Our analysis revealed a significant decrease in the total dendritic length (Figure 5B, F), number of branching points (Figure 5C, G), and intersections (Figure 5D, H) of DCX-positive cells in both short-term and long-term of QCM-TBI mice compared to control mice. However, we did not observe significant alterations in the process of IBA-1- and GFAP-positive cells of QCM-TBI mice compared to control mice (Figure S7). QCM-TBI mice showed neuronal damage, including synaptic and axonal injury, which were associated with changes in the hippocampal environment affecting adult neurogenesis. Markers such as BrdU, DCX, NeuroD1, and NeuN were used to track neuronal development, while GFAP, IBA-1, and OLIG2 were used to assess astrocytes, microglia, and oligodendrocytes, respectively (Figure 5I).

### QCM-TBI induces force-dependent neuroinflammation and cerebrovascular injury

To investigate the transcriptional regulation of inflammatory processes following QCM-TBI, we performed Gene Ontology (GO) enrichment analysis on upregulated DEGs in the hippocampus of F20 and F40 mice compared to controls. The GO terms enriched in biological process (BP), molecular function (MF), and cellular component (CC) categories revealed significant enrichment of immune-related pathways, including inflammatory response, cytokine activity, and leukocyte activation (Figure S4A-C). Bar plots illustrating DEG expression patterns (Figure S4D-E) showed a dose-dependent increase in proinflammatory gene expression between F20 and F40 groups. A Venn diagram (Figure S4F) demonstrated that 9 genes were commonly upregulated in both QCM-TBI groups. Among these, *Cxcl16*, *Il17re*, and *Il1a* exhibited robust force-dependent upregulation (Figure S4G), which was further validated by qRT-PCR analysis in dorsal hippocampus of QCM-TBI mice (Figure S4H). These findings suggest that QCM-TBI induces a graded neuroinflammatory response in the hippocampus that scales with impact severity.

To assess the impact of QCM-TBI on cerebrovascular integrity, we examined the expression of platelet-derived growth factor receptor beta (PDGFRβ), a pericyte-associated vascular marker, in mouse brain tissue. Immunohistochemical analysis revealed a significant reduction in PDGFRβ expression in the SCx, DG and CA3 region of QCM-TBI mice compared to controls (Figure S11A-B). Densitometric quantification confirmed these decreases, suggesting vascular damage and potential blood-brain barrier (BBB) disruption following traumatic impact. Consistently, in human CTE patient cortex, PDGFRβ expression in blood vessels was also markedly decreased (Figure S11D-E), supporting the translational relevance of QCM-TBI-induced vascular pathology. These data indicate that QCM-TBI compromises cerebrovascular structure in a manner analogous to that observed in chronic human TBI pathology.

### QCM-TBI induces force-dependent neuropathological changes resembling CTE in mice

**Figure 6:**
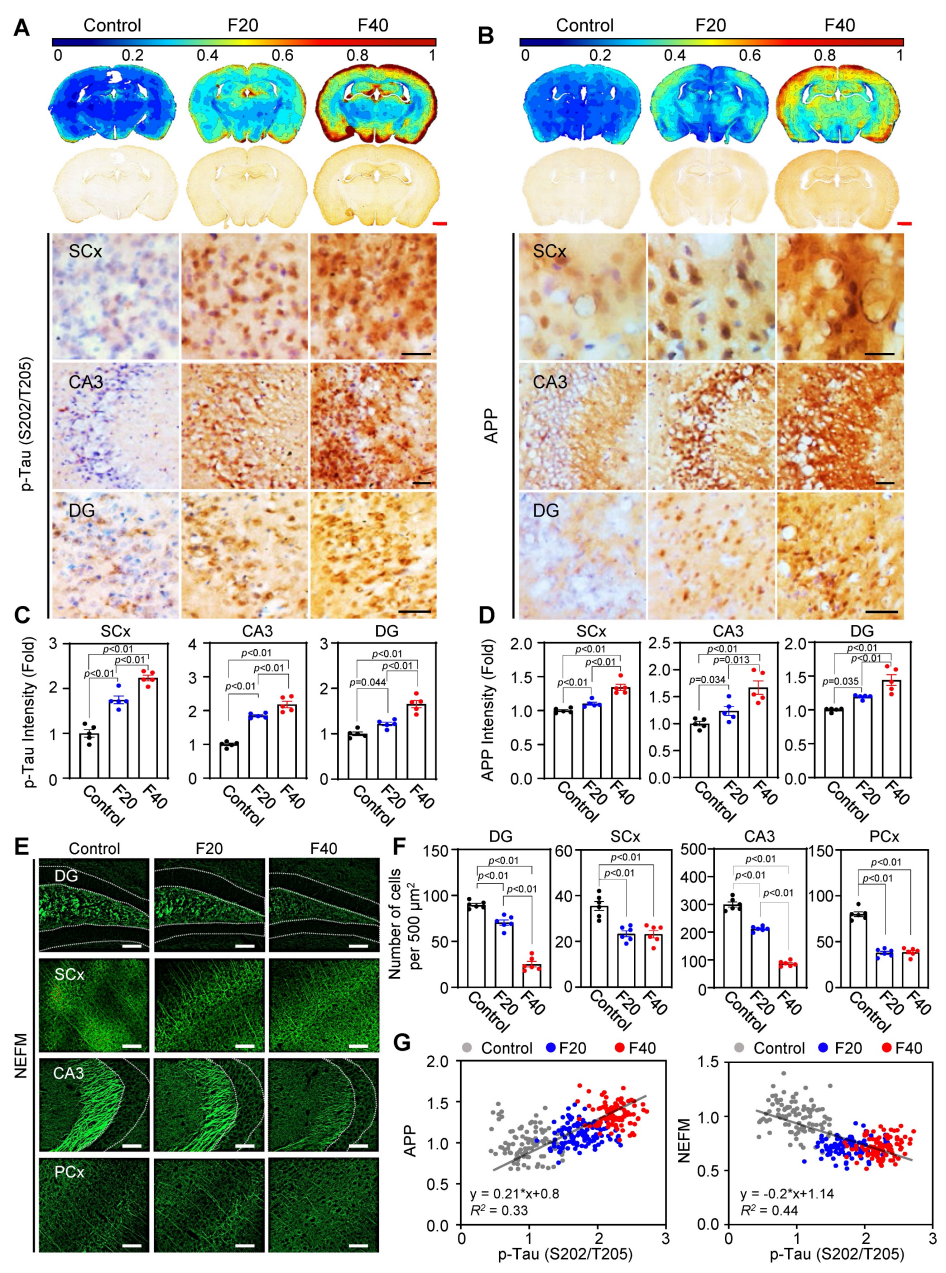
Tau phosphorylation and APP levels are increased but NEFM level is decreased in QCM-TBI mice. **A, C** Immunoreactivity of phosphorylated p-Tau (S202/T205) was increased by QCM-TBI. Densitometry analysis showed that p-Tau (S202/T205) is significantly elevated in the SCx, CA3 and the dentate gyrus (DG) of the QCM-TBI-induced mouse brain in an impact force-dependent manner. Scale bars: 1 mm (*red*); 50 µm (*black*). Contour map (blue color, lower intensity; red color, higher intensity). **B, D** Immunoreactivity of amyloid precursor protein (APP) was increased by QCM-TBI. The densitometry analysis showed that APP was significantly increased in the SCx, CA3 and DG of QCM-TBI-induced mouse brains in an impact force-dependent manner. Scale bars: 1 mm (*red*); 50 µm (*black*). A total of 50 to 100 cells were examined from *n* = 5. **E** Immunostaining showing the expression of NEFM in control, F20 and F40 QCM-TBI conditions. Scale bars: 100 µm. **F** Bar graph demonstrates the number of immuno-positive cells in each condition (control, *n* = 6; F20, *n* = 6; F40, *n* = 6). **G** Relative fold changes in APP and NEFM expression levels compared with AT8 expression in QCM-TBI-induced models. Dot plot represents the gene expression level in QCM-TBI models of each condition with respect to the changes in APP and NEFM with AT8 expression. A total of 100 cells (20 cells/case) were examined from *n* = 5 mice. Data represents the mean ± SEM.

Previous studies have demonstrated that mechanical impacts from QCM-TBI strongly affect neuronal gene expression and may be correlated with pathological changes in mature neurons 20, 22, 25. To further investigate this possibility, we analyzed neuropathological changes by examining the levels of p-Tau (S202/T205), APP, and NEFM using IHC analysis (Figure 6). Our contour map analysis revealed that p-Tau (S202/T205) immunoreactivity, a pathological marker of TBI, increased throughout the whole brain, particularly in the SCx, CA3 and DG regions, in an impact-dependent manner following QCM-TBI (Figure 6A, C). In addition, APP immunoreactivity, a marker of axonal damage, was significantly elevated in the SCx, CA3 and DG regions, also in an impact-dependent manner following QCM-TBI (Figure 6B, D). Moreover, the levels of NEFM were significantly decreased in the DG, SCx, CA3, and PCx regions of QCM-TBI mice, particularly at F40 (Figure 6E-F and Figure S14A-B). Interestingly, we observed a positive correlation between the increase in p-Tau (S202/T205) and APP levels in an impact-dependent manner following QCM-TBI (Figure 6G). In contrast, the levels of NEFM showed an inverse correlation with impact, suggesting that these pathological changes resemble the phenotype of CTE patients. We also examined the levels of p-Tau (S396 and S199) (Figure S12A-D), neurofilament heavy polypeptide (NEFH) and neurofilament light polypeptide (NEFL) (Figure S14C-F) following QCM-TBI. These findings suggest that pathological changes and axonal damage are affected in a force-dependent manner by QCM-TBI.

To assess region-specific tau pathology following QCM-TBI, we examined the expression of phosphorylated tau epitopes in the basolateral amygdala (BLA). p-Tau (S202/T205) levels were significantly increased in the BLA of QCM-TBI mice compared to controls (Figure S12E-F). In contrast, p-Tau (S396 and S199) levels did not exhibit measurable changes in this region. These findings suggest that tau phosphorylation in the BLA may occur in a site-specific manner and highlight the regional vulnerability and heterogeneity of tau pathology induced by mechanical brain injury. To investigate the regional effects of single and repeated mechanical impacts (F40) on tau phosphorylation and myelination, we analyzed p-Tau (S202/T205) and myelin basic protein (MBP) expression across multiple brain areas in QCM-TBI mice. Interestingly, single QCM-TBI induced a significant increase in p-Tau levels in dentate gyrus (DG), CA3, and basolateral amygdala (BLA). Repeated QCM-TBI induced a significant, frequency-dependent increase in p-Tau levels in the somatosensory cortex (SCx), DG, CA3, and BLA, whereas the thalamus (TH) showed no substantial change (Figure S8). Similarly, MBP expression was markedly reduced in the SCx, corpus callosum (CC), DG, and CA3, indicating region-specific demyelination, while TH and BLA remained unaffected (Figure S9). These results suggest that repeated QCM-TBI induces selective tau pathology and white matter disruption in brain regions vulnerable to the impact of mechanical stress, potentially contributing to the progression of TBI-related neurodegeneration.

To evaluate pathological similarities between QCM-TBI mice and human chronic traumatic encephalopathy (CTE), we assessed p-Tau and APP expressions in both models. In CTE patient cortex, immunohistochemistry revealed marked accumulation of p-Tau at multiple phosphorylation sites, including S202/T205, S396, and S199 (Figure S13A). Correspondingly, QCM-TBI mice also showed significant upregulation of p-Tau at the same epitopes, as confirmed by densitometric analysis (Figure S13B), indicating conserved patterns of tau hyperphosphorylation across species. In addition, APP immunoreactivity was elevated in the cortex of CTE patients, particularly in both gray and white matter compartments (Figure S13C-D). These findings support the notion that QCM-TBI recapitulates key molecular hallmarks of human CTE, including tauopathy and axonal injury-associated APP accumulation.

### QCM-TBI induces axonal damage and alters nuclear morphology

**Figure 7:**
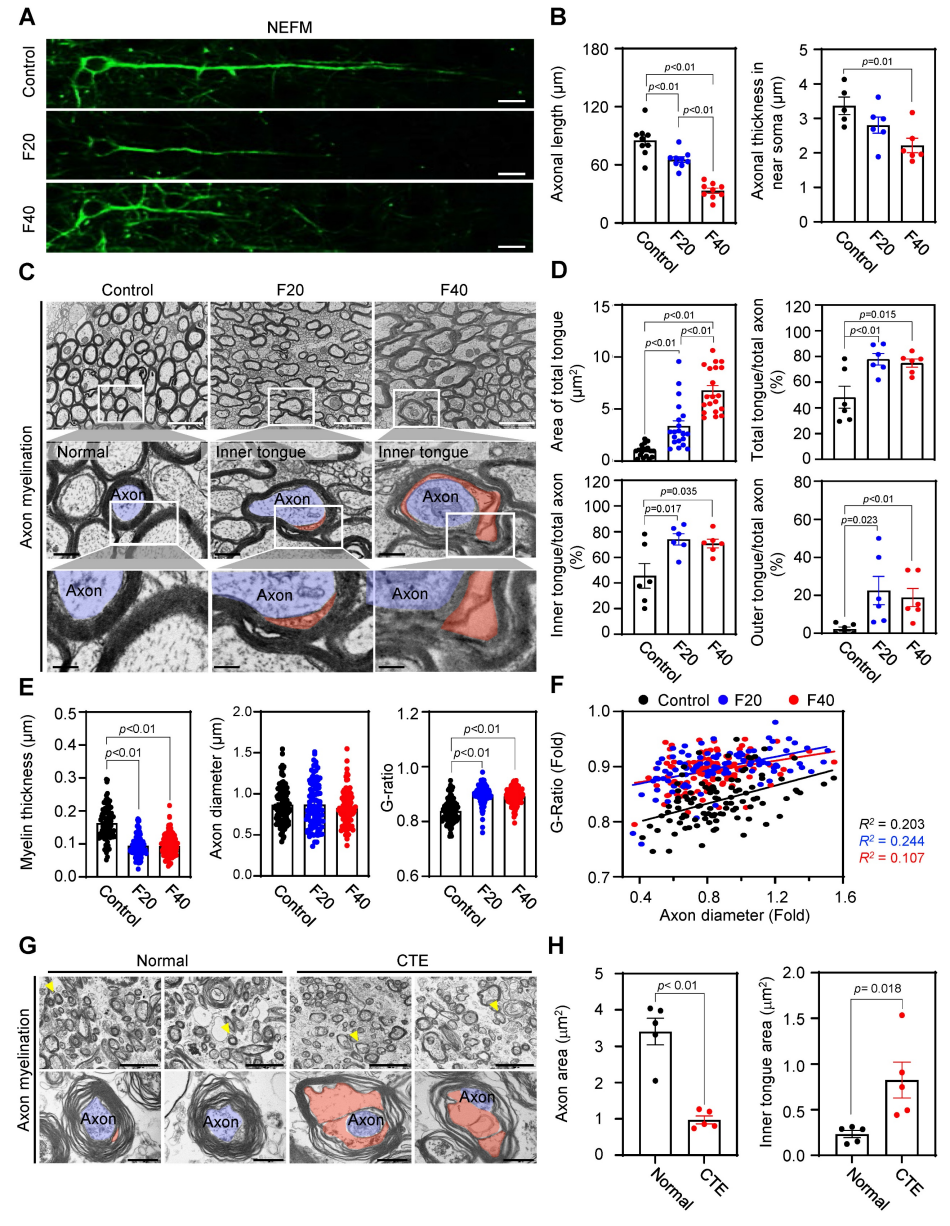
Axonal length and myelin sheath structure are affected by QCM-TBI. **A** Representative image of NEFM-positive neurons in each condition (control, F20 and F40). Scale bars: 10 µm. **B** Bar graph showing the length and thickness of axons in each condition (control, F20 and F40). A total of 9 cells were examined from *n* = 3. **C** Axonal ultrastructure was altered in the corpus callosum by QCM-TBI. Scale bar: 2 µm (*white and black*). **D** Relative size and number of the inner and outer tongue area were altered in the corpus callosum by QCM-TBI. **E** Myelin thickness and axonal diameter were decreased by QCM-TBI. (control, examination of a total of 101 axons; F20, a total of 97 axons; F40, a total of 111 axons). **F** The G-ratio was significantly altered by QCM-TBI. **G** Axonal ultrastructure was altered in the cortex of CTE patient. Scale bar: 5 µm (*upper*), 0.5 μm (*lower*). **H** Relative size and number of the axon area and inner tongue area were altered in the cortex of CTE patients. Data represents the mean ± SEM.

We found that the length and thickness of NEFM-positive axons were significantly decreased in the CC of QCM-TBI mice (Figure 7A-B). Using TEM imaging, we further analyzed ultrastructural changes in neuronal axons and found that myelin thickness was significantly reduced (Figure 7E-F), while the G-ratio was significantly increased in the CC of QCM-TBI mice (Figure 7E-F). Moreover, we identified an increase in the total area of tongue and the number of outer and inner tongue of axon myelination in the corpus callosum of QCM-TBI mice (Figure 7C-D). In the CTE cortex, axons exhibited marked morphological abnormalities, including disrupted myelin compaction and distorted axoplasmic architecture (Figure 7G). Consistently, QCM-TBI mice displayed significant alterations in axonal ultrastructure, particularly within the corpus callosum. Quantitative analysis revealed that both the axon area and the inner tongue area of the myelin sheath were significantly changed compared to controls (Figure 7H), suggesting demyelination and impaired axonal integrity. These findings demonstrate that QCM-TBI induces ultrastructural axonal pathology comparable to that observed in human CTE brains. To further assess myelin integrity following QCM-TBI, we examined MBP expression in brain regions associated with cognitive and sensorimotor function. QCM-TBI mice exhibited a notable reduction in MBP immunoreactivity in the SCx, DG, and CA3 region compared to control (Figure S10). This regional loss of MBP suggests that traumatic impact disrupts myelin structure in functionally critical areas of the brain, potentially contributing to deficits in neural connectivity and cognitive performance observed in QCM-TBI mice.

In addition to axonal damage, we also analyzed changes in soma size by cresyl violet (CV) staining in SCx and nuclear structure by Lamin B1 staining and TEM imaging in the CC region of QCM-TBI mice (Figure S15A-C). We found that the cell size was decreased and corrupted nuclear morphologies were detected in the F20 and F40 of QCM-TBI mice, respectively (Figure S15A-B). We also analyzed the nuclear aspect ratio (length/width), roundness, and circularity (Figure S15C-D) and found that QCM-TBI significantly increased the aspect ratio but decreased the roundness and circularity of the nuclear envelope (Figure S15D). These data suggest that neuropathological changes caused by QCM-TBI are more strongly related to axonal damage than nuclear structure. Our study provides detailed insights into the effects of QCM-TBI on axonal damage, which may have important implications for understanding the pathophysiology of TBI.

### Effect of QCM-TBI on wake-up time, locomotor behaviors and cognitive functions

**Figure 8:**
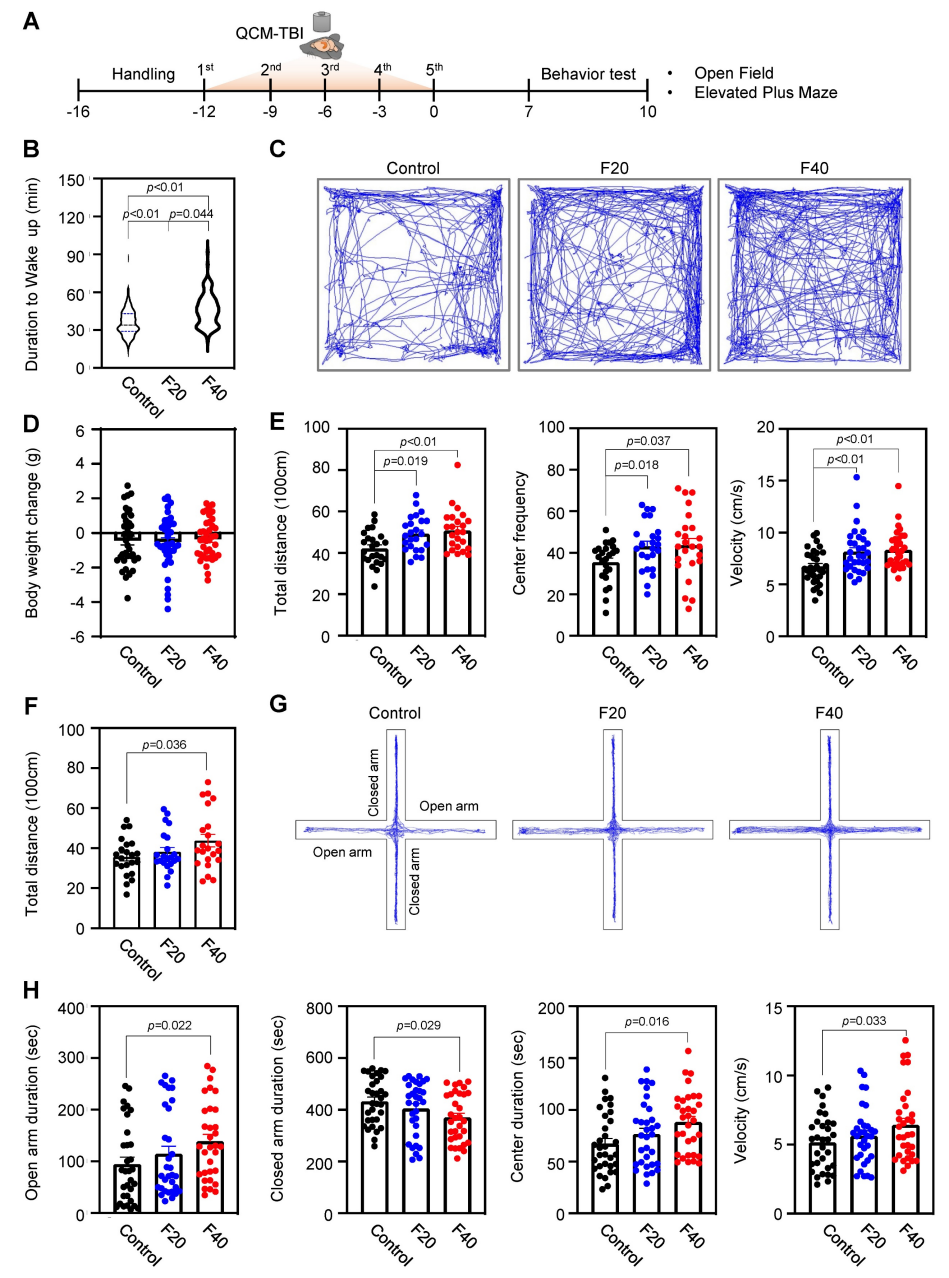
The duration to wake up and locomotive behaviors are affected in QCM-TBI mice. **A** Experimental scheme of QCM-TBI for behavioral test. **B** QCM-TBI significantly delayed the duration of waking up in a force-dependent manner (control, *n* = 27; F20, *n* = 30; F40, *n* = 27). **C** Representative images of total moving traces of QCM-TBI mice in the open field test (OFT). **D** There were no significant body weight changes between the control and the QCM-TBI groups of mice (control, *n* = 38; F20, *n* = 41; F40, *n* = 39). **E,** Total distance, center frequency, and velocity of movement in the OFT were significantly elevated in QCM-TBI mice compared to control mice (control, *n* = 24; F20, *n* =24; F40, *n* = 24). **F** Total distance of movement in the elevated plus maze (EPM). **G** Representative images of total moving traces of QCM-TBI mice in the EPM. **H** Open arm duration, center duration, and velocity in the EPM were significantly elevated while closed arm duration was reduced in QCM-TBI mice compared to control mice (control, *n* = 32; F20, *n* = 32; F40, *n* =32). Data represent the mean ± SEM.

To clarify the relationship between TBI-induced mechanical impacts and behavioral outcomes, we conducted a comprehensive analysis of wake-up time and locomotor behaviors in QCM-TBI mice. Our findings reveal that wake-up time was significantly delayed in an impact-dependent manner following QCM-TBI (Figure 8A-B). We also examined the effects of QCM-TBI on mouse behavioral parameters and interestingly observed that overall locomotor activity in the open field test (OFT) was increased in QCM-TBI mice compared to control mice (Figure 8C-E). Specifically, the total distance and velocity of movement were significantly higher in QCM-TBI mice than in control mice (Figure 8E). Furthermore, the time spent in the center of the elevated plus-maze (EPM) was significantly increased in QCM-TBI mice compared to control mice (Figure 8F-H), indicating increased anxiety-like behavior in QCM-TBI mice. It is worth noting that there were no significant changes in body weight between the three groups of mice subjected to QCM-TBI (Figure 8D). Taken together, our results suggest that mechanical impacts of TBI can lead to delayed wake-up time and altered locomotor and anxiety-related behaviors in an impact-dependent manner, highlighting the need for further investigation into the impact of TBI on behavioral outcomes.

To assess cognitive impairments induced by QCM-TBI, we employed NOR and NOPR tests, as illustrated in the experimental scheme (Figure S16A-B). These paradigms evaluate object recognition memory and spatial memory, respectively, which are commonly affected by traumatic brain injury. Representative heatmaps of exploration behavior (Figure S16C) revealed reduced investigation time around the novel object and its relocated position in QCM-TBI mice. Quantitative analysis demonstrated a significant decrease in the discrimination index in both NOR (Figure S16D) and NOPR (Figure S16E) tasks in QCM-TBI mice compared to controls. These findings indicate that the modified QCM-TBI model leads to long-term deficits in both recognition and spatial memory, reflecting cognitive impairments relevant to clinical TBI pathology.

## Discussion

Despite numerous animal models of TBI being utilized to investigate the underlying mechanisms of closed-brain injury, the development of engineered and mechanistic animal models that closely mimic human closed-brain injury remains challenging 17, 19, 23, 24. In contrast, our QCM-TBI model, a reliable engineered diffuse closed-brain injury model, overcomes several limitations and offers distinct benefits when compared to existing animal models of TBI, as outlined below. In a previous study, rats were used to investigate the correlation between mechanical impact magnitude and neurological dysfunctions, requiring external devices to be mounted on the animal's head to estimate force delivery 22. However, this approach introduced technical challenges, including limited precision and variability in head fixation. In contrast, our study utilized a mouse-based QCM-TBI model equipped with an integrated high-resolution sensor system that enables direct and quantitative measurement of mechanical force delivered to the brain. This design not only eliminates the need for invasive head-mounted devices but also allows for reproducible application of graded and repetitive impacts, with real-time recording of force dynamics at the moment of collision (Figure 1-2).

Compared to traditional TBI models such as controlled cortical impact (CCI) and weight-drop injury, the QCM-TBI system provides a transformative advantage through real-time force quantification, offering a quantitative framework for correlating mechanical loading with molecular, cellular, and behavioral outcomes. Prior models have lacked precise impact control, limiting their ability to define threshold forces that initiate axonal injury or tau pathology 2, 51. Our findings demonstrate that force-dependent pathology is not only reproducible but also mechanistically relevant to human TBI signatures, including APP deposition and tau phosphorylation, which have been implicated in long-term neurodegenerative risk 11, 52. This advancement enables preclinical studies to move beyond descriptive endpoints toward predictive modeling of injury severity and therapeutic responsiveness.

We utilized a sensor-fusion and optimization technique, which had not been possible previously due to the highly accelerating sensor's distorted baseline (Figure 1-2). This new technique enabled us to accurately quantify the amount of resulting impact, as well as investigate a transient force profile over the time of collision. In contrast, previous TBI animal models have been limited to indirect parameters such as the velocity of colliding objects or the head acceleration of the animal, hindering accurate quantification of impact 22, 25, 26. Therefore, our QCM-TBI model provides a significant improvement over previous animal models of TBI, allowing for more precise and accurate measurements of the mechanical impact delivered to the brain 20, 21, 34.

The QCM-TBI model has a gravity compensating support that allows animals to move naturally, resulting in translational and rotational acceleration based on the nature and severity of impact 20, 22, 25, 26, 34. Because both humans and animals have a flexible spine structure, head movement through cervical flexion and extension produces coup and countercoup effects in response to different levels of impact (Figure S1). Consequently, the QCM-TBI model with engineered animal support could be an optimal solution to allow the natural body motion of animals during TBI experiments under anesthesia. To validate the contribution of the gravity-compensating animal support, we compared its performance in quantitative TBI measurement with a simple spring support possessing the same opening structure in place of the gravity compensation mechanism (Figure 1-2). Interestingly, the proportional relationship between weight and net impulse holds only with the gravity compensation mechanism for all ranges of applying velocities, while inconsistent results were obtained with the simple spring support, specifically for low impact (Figure 1-2). This inconsistency occurs because the simple spring installment imposes an unavoidable extra force to initially support the animal and to open the supporting plates during collision, which affects the calculation of net impulse. This undesirable effect becomes prominent as the portion of intact force led by collision reduces with low impact. Furthermore, our QCM-TBI model addresses the issue caused by the rebound effect that inevitably occurs in weight-drop models. It also prevents the severe head injury observed in other models that used foam pads for animal support 20, 21, 38, and the cervical flexion/extension potentially mitigates the amount of delivered impact.

To capture transcriptional responses that scale with mechanical impact, we conducted a force-resolved analysis of gene expression across graded QCM-TBI conditions. Unlike conventional DEG approaches that rely on binary group comparisons (e.g., injured vs. sham), our analysis introduced the concept of force-dependent genes (FDGs), defined by linear regression of expression values across increasing net impulse levels. Genes with a slope magnitude greater than 0.1 and R² exceeding 0.8 were classified as FDGs, revealing a distinct subset of genes whose expression changed in a graded, dose-responsive manner. This approach allowed us to distinguish genes truly sensitive to biomechanical severity, such as *Dcx*, *NeuroD1*, *Syn1 and Map2* which were validated via qRT-PCR and exhibited progressive downregulation with increasing force. Similarly, Fth1, an iron metabolism-related gene, showed consistent force-dependent regulation at both transcriptomic and protein levels. These findings support the notion that specific molecular pathways-especially those involved in neurogenesis, iron handling, and inflammatory signaling-are mechanosensitive and scale proportionally with TBI severity. By implementing this force-resolved transcriptomic framework, we provide a novel strategy to characterize the biological gradient of closed-head injury, which may be useful in linking preclinical models with clinical injury spectra.

Although several studies have been conducted, there is still a lack of clarity regarding which brain cell types are most affected by the physical impact in TBI animal models 20, 22, 25, 26, 38. To address this knowledge gap and identify the direct effects of mechanical impacts on various brain cell types, we performed RNA sequencing analysis on brain samples from QCM-TBI mice and compared the results with transcriptome data from postmortem brain samples of CTE patients (Figure 3) 53. Through this analysis of cell-type-specific transcriptome signatures in QCM-TBI and CTE, we discovered that neuronal-specific genes were significantly down-regulated in QCM-TBI mice and CTE patients 54-56, as compared to non-neuronal cell types such as astrocytes, microglia, and oligodendrocytes (Figure S6). This finding may suggest that axonal damage to neuronal cells is more sensitive to mechanical damage than non-neuronal cells. Previous studies have reported contradictory findings regarding alterations to adult neurogenesis in the DG of TBI animal models and CTE patients 57. However, our study has revealed that genes related to adult hippocampal neurogenesis were consistently down-regulated in both QCM-TBI mice and CTE patients (Figure 3-5). Specifically, we observed a reduction in the expression of DCX, NeuroD1, and NeuN genes, which are indicative of disrupted neurogenesis in QCM-TBI and CTE 63. These findings are consistent with the reported loss of memory and learning ability in both TBI animal models and CTE patients 58-61. Notably, the down-regulation of NeuroD1 in QCM-TBI and CTE suggests a disturbance in neuronal differentiation and specification in the hippocampus 62, 63. We further validated this finding by analyzing the neurite outgrowth of DCX-positive cells, which demonstrated a decrease in NeuroD1 levels and differentiation of immature neurons in QCM-TBI mice compared to control mice (Figure 4-5). Therefore, our study supports the notion that TBI deregulates neurogenesis- and neuronal differentiation-associated gene signatures in a cell-type specific manner.

Previous studies have shown that TBI can lead to dysregulation of adult neurogenesis 56, 64. However, it remains unclear whether this dysregulation persists over a long-term period following TBI. In our study, we demonstrated that the maturation of immature neurons decreased both in the short-term (7 days of post-TBI) and long-term (120 days of post-TBI) effects on QCM-TBI (Figure 4-5). These findings suggest that the continuous impact of mature neuronal damage, such as p-Tau and axonal damage in the DG, may have ongoing effects on neuronal stem cells in DG of QCM-TBI mice compared to control mice. Interestingly, TBI also damages mature neurons in the cortex and hippocampus. Our QCM-TBI animal model exhibits neuropathological features, including increased p-Tau and amyloidosis, and shortened neurofilament length, that are correlated with the applied impact. Repetitive head injuries induced by QCM-TBI system increased p-Tau levels in neurons 65-68, which is comparable to human CTE studies that have shown elevated p-Tau levels and diffuse senile plaques in the postmortem brains of CTE patients 12-16. In addition, QCM-TBI model revealed that the levels of p-Tau (S202/T205) and APP were elevated in an impact-dependent manner. Notably, elevated APP levels were localized in axonal arborizations, areas where the level of p-Tau was also increased in QCM-TBI mice (Figure 6). Notably, similar patterns of axonal and myelin pathology have been reported in human postmortem studies of CTE, in which repetitive brain trauma leads to axonal degeneration, p-Tau accumulation, and white matter loss. Although classical APP-positive axonal varicosities are rare in chronic-stage human CTE, overall APP immunoreactivity and myelin abnormalities remain prominent. Thus, the pathological phenotypes induced by QCM-TBI recapitulate key features of early-stage CTE and support the translational utility of this model in studying trauma-induced neurodegeneration.

Previous studies have found that APP induction by TBI is linked to decreased numbers of neuronal precursor cells and immature neurons in the dentate gyrus, followed by constant neuronal loss in CA3 regions 54. Based on these results, we propose that changes in APP levels due to TBI may have a significant effect on neurons, including neuronal precursor cells, immature neurons, and mature neurons. In contrast to the increase in p-Tau levels, our QCM-TBI model resulted in a reduction of neurofilament length and intensity, which is also observed in the neuropathology of CTE 20, 51. Neurofilaments (NFs) are neuron-specific intermediate filaments that play a crucial role in regulating axonal growth and maintaining neuronal homeostasis 52, 69. Alterations in the levels of NFs in serum or cerebrospinal fluid (CSF) serve as important markers of neurodegeneration in TBI and AD 70, 71. It is likely that the degradation and secretion of NFs into the CSF and bloodstream reflect the status of mature neuron damage and neurodegeneration in the brain 72. NFs consist of three subunits (NEFL, NEFM, and NEFH), and the expression level of each subunit plays a critical role in maintaining healthy neurons. NFs interact with microtubules and microfilaments to maintain axonal integrity and neuronal cytoskeleton 70, 73. Our findings revealed reduced NFs expression in the cortex and hippocampus without overt retraction bulbs or inflammatory nodules, suggesting early-stage axonal injury. This is consistent with prior reports showing that neurofilament disruption can precede axonal fragmentation and may reflect cytoskeletal destabilization under mechanical and metabolic stress 74, 75. The absence of strong inflammatory infiltration within 7 days post-injury aligns with the known delayed immune response characteristic of diffuse axonal injury models 76-78. Furthermore, our previous study showed that increased p-Tau levels lead to neurofilament tangle formation in CTE 79. The p-Tau reduces the binding of NFs to microtubules, causing their dissociation from neuronal microtubules, and ultimately generating neurofibrillary tangles in neuronal cells, leading to neuronal dysfunction and neurotoxicity in neurodegenerative diseases 80-82. Therefore, our results suggest that the degradation of NEFM and p-Tau has a significant impact on neuronal activity in QCM-TBI. However, the molecular and cellular mechanisms underlying the degradation and release of NFs remain to be fully elucidated in future studies (Figure 6-7).

Moreover, numerous studies in both animal models and human postmortem brains have demonstrated that the hippocampus exhibits marked neuronal loss, gliosis, and tau pathology in response to both acute and repetitive injuries 14-16. In CTE patients, hippocampal degeneration-including CA1 shrinkage, dentate granule cell loss, and subpial tau deposition-is consistently observed and correlates with cognitive dysfunction and memory decline. Thus, focusing our transcriptome analysis on the hippocampus enabled us to interrogate molecular mechanisms related to neurogenesis, synaptic integrity, and inflammatory signaling in a region that is pathologically and functionally central to both TBI and CTE. Future studies will expand spatial coverage to other regions such as the prefrontal cortex and thalamus to further define the regional transcriptomic landscape in QCM-TBI.

Adult neurogenesis is increasingly recognized as a process that can be dysregulated in various pathological conditions, including neuroinflammation, neurodegeneration, and psychiatric disorders 55, 83, 84. In the context of TBI, the hippocampus is particularly vulnerable, and TBI has been shown to induce complex changes in the neurogenic niche. These alterations contribute to injury-induced maladaptive plasticity or compensatory repair mechanisms. In this context, to provide a valuable window into the cellular and molecular pathological consequences of injury and understand mechanisms of neuropathological dysfunction by the QCM-TBI model, we investigated the alteration of adult neurogenesis in our system.

In this study, we observed force-dependent accumulation of p-Tau and APP in the QCM TBI model, consistent with prior reports of acute tau phosphorylation following TBI in both experimental and human contexts 65, 85-87. Notably, plasma and CSF p-Tau levels rise within hours after acute TBI in humans, reinforcing tau phosphorylation as an early mechanistic event rather than a delayed chronic marker 87, 88. Importantly, our interpretation does not assert a clinical diagnosis of chronic traumatic encephalopathy (CTE); instead, we frame these findings as mechanistic: acute, force-dependent tau phosphorylation may contribute to downstream tauopathies. Similarly, the transient post-impact anesthesia observed in rodents—quantified via righting reflex recovery—is presented solely as a functional measure of procedural recovery kinetics, not as a surrogate for human coma. Finally, we took rigorous care to distinguish glial versus neuronal structures: MAP2 (neuronal dendrites), GFAP (astrocytes), and IBA-1 (microglia) were used to specifically attribute cellular localization in our immunohistochemistry 66, 89, thereby avoiding conflation of glial processes with neuronal neurites. Collectively, these clarifications highlight that QCM TBI offers a rigorously controlled preclinical platform for dissecting acute injury cascades, effectively bridging biomechanical impact to neuropathological changes relevant to human TBI.

Prior works have reported that TBI can cause changes in locomotor and emotional behavior in animal models and patients 90-95. Although the present study did not incorporate the Neurological Severity Score (NSS), we assessed behavioral outcomes using a set of validated assays, including latency to righting reflex (LRR) for acute consciousness impairment, open field test (OFT) for locomotor behavior, and elevated plus maze (EPM) for anxiety-related responses. These tests were chosen to capture functionally relevant endpoints in a force-dependent manner and are widely used in TBI models for characterizing early and subacute behavioral changes. Nevertheless, we acknowledge that NSS provides a structured and comprehensive assessment of post-traumatic neurological function, including reflexes, motor coordination, and balance 96. Incorporating NSS in future studies will allow for a more integrative assessment of QCM-TBI severity and its correlation with neuropathological and molecular outcomes across impact intensities. In our study, we found that the duration of wake-up after QCM-TBI was delayed in an impact-dependent manner, suggesting that the severity of the injury may affect the duration of coma status in mice. While there is a correlation between the coma scale and morbidity and mortality in human TBI patients, predicting the degree of impact and coma status is not feasible 97-99. Therefore, our QCM-TBI animal model can be useful in studying the correlation between different impact levels and coma status and neuropathology (Figure 8). Additionally, QCM-TBI mice showed significantly increased locomotor activity in the OFT. Specifically, the total distance of movement and velocity were significantly elevated, and these changes correlated linearly with impact levels in QCM-TBI mice when compared to control mice. While the present study focused primarily on neuropathological and transcriptomic signatures, we also evaluated memory performance using the novel object recognition (NOR) test in a long-term version of the QCM-TBI model (Figure S16). F40 group of QCM-TBI exhibited a significantly reduced discrimination index compared to controls, suggesting impaired recognition memory by novel object recognition (NOR) and novel object place recognition (NOPR) test. These findings are consistent with the notion that force-dependent closed-head TBI leads not only to suppressed neurogenesis and synaptic alterations in the hippocampus, but also to long-term cognitive dysfunction. Our QCM-TBI animal model provides the first evidence that behavioral and neuropathological changes are correlated with the quantitative impact of brain injury. From a translational perspective, the QCM-TBI platform offers a unique opportunity to systematically evaluate candidate neuroprotective interventions under controlled mechanical conditions. By enabling precise dose-response studies, this model can identify critical windows for therapeutic intervention and assess treatment efficacy in a manner that more closely reflects clinical variability in TBI severity. Additionally, early biomarkers identified in this model—such as p-Tau and APP—may serve as molecular readouts for treatment response, accelerating bench-to-bedside translation. Ultimately, these capabilities position QCM-TBI as a foundational tool for designing and validating targeted therapies aimed at reducing the long-term burden of TBI-related neurodegeneration.

## Conclusions

In conclusion, we have developed a novel apparatus and methodology, called QCM-TBI, which enables us to create an animal model of closed-brain injury with higher precision and reproducibility. By measuring the instantaneous force change over the time of collision, QCM-TBI allows accurate assessment of the net impulse. Moreover, it can replicate cervical flexion/extension in vertebrae during impact, thanks to the use of gravity-compensating animal support. Our study has also shown that TBI leads to the deregulation of adult neurogenesis-associated transcriptome signatures in an impact-dependent manner, highlighting its pathological consequences. Furthermore, the levels of APP, p-Tau and NEFM are strongly associated with neuronal activity in QCM-TBI. Taken together, our findings suggest that TBI not only induces neuropathological changes but also causes abnormal animal behavior depending on the force of the impact, leading to a significant reduction in quality of life. We anticipate that our QCM-TBI model will help to shed light on the causal relationship between physical impact and neuropathological and behavioral consequences of repetitive head injury, thus paving the way for new treatments for TBI.

## Supplementary Material

Supplementary figures and tables.

Supplementary additional file: alignment statistics.

Supplementary additional file: total gene expression.

Supplementary additional file: differentially expressed genes of QCM-TBI.

Supplementary additional file: the FPKM levels of each gene.

## Abbreviations

TBI: Traumatic brain injury
QCM-TBI: Quantitatively Controlled and Measured-TBI
CTE: Chronic traumatic encephalopathy
p-Tau: Phosphorylated Tau
APP: Amyloid precursor protein
AD: Alzheimer's disease
IACUC: Institutional Animal Care and Use Committee
DEGs: Differentially expressed genes
ENA: European Nucleotide Archive
GO: Gene ontology
FPKM: Fragments Per Kilobase Million
qRT-PCR: Quantitative RT-PCR
OFT: Open field test
EPM: Elevated plus maze
LRR: Latency to righting reflex
TEM: Transmission electron microscopy
FT: Force-time
FDGs: Force-dependent genes
Fth1: Ferritin heavy chain 1
DG: Dentate gyrus
CA: Cornu ammonis
PCx: Pyriform cortex
SNT: Simple Neurite Trace
DCX: Doublecortin
BrdU: 5-bromo-2-deoxyuridine
NeuroD1: Neurogenic differentiation 1
NeuN: A mature neuronal marker
GFAP: Glial fibrillary acidic protein
OLIG2: Oligodendrocyte transcription factor 2
IBA-1: Allograft inflammatory factor 1
SCx: Somatosensory cortex
NEFM: Neurofilament medium polypeptide
NEFH: Neurofilament heavy polypeptide
NEFL: Neurofilament light polypeptide
CV: Cresyl violet
OFT: Open field test
EPM: Elevated plus-maze
BLA: Basolateral amygdala
CC: Corpus callosum
TH: Thalamus
