# Supplementary material for: Quantitatively controlled and measured-traumatic brain injury impairs adult neurogenesis and alters neuropathological signatures in mice
Source: Theranostics. 2026 Jan 1;16(1):193–219. doi: 10.7150/thno.114693 (PMC12665107; doi:10.7150/thno.114693)
Supplement: Supplementary file 1 — Supplementary figures and tables. [file thnov16p0193s1.pdf]

Supplementary Information

Quantitatively Controlled and Measured-Traumatic Brain Injury Reveals Force-Dependent Impairment of Adult Neurogenesis and Neuropathological Mechanisms

Sungwook Yang[1#], Suhyun Kim[2#], Uiyeol Park[2,9#], Hyeonjoo Im[2,3], Hyesun Cho[2], Kyung Eun Lee[4], Junsang Yoo[2], Seung Jae Hyeon[2], Se Jeong Lee[3], Im Joo Rhyu[3], Junghee Lee[5,6], Ann C. McKee[5,6], Eui-Sung Yoon[7]*, Hoon Ryu[2,8]*

[1]Center for Humanoid Research, Artificial Intelligence and Robot Institute, Korea Institute of Science and Technology (KIST), Seoul 02792, Republic of Korea

[2]Laboratoy for Brain Gene Regulation and Epigenetics, Center for Brain Disorders, Brain Science Institute, KIST, Seoul 02792, Republic of Korea

[3]Department of Anatomy, College of Medicine, Korea University, Seoul 20841, Republic of Korea

[4]Advanced Analysis Data Center, KIST, Seoul 02792, Republic of Korea

[5]Boston University Alzheimer's Disease Research Center and Department of Neurology, Boston University School of Medicine, Boston, MA 02118, USA

[6]VA Boston Healthcare System, Boston, MA 02130, USA

[7]Center for Brain Convergence Research, Brain Science Institute, KIST, Seoul 02792, Republic of Korea

[8]KHU-KIST Department of Converging Science and Technology, Kyung Hee University, Seoul 02447, Republic of Korea

[9]Department of Medicine, Hanyang University Medical School, Seoul 04763, Republic of Korea

[#]These authors are equal contributors.

[*]Corresponding authors

Eui-Sung Yoon, Ph.D. E-mail: esyoon@kist.re.kr

Hoon Ryu, Ph.D. E-mail: hoonryu@kist.re.kr

Supplementary Table

Table S1 List of qRT-PCR primer sequence.

Model	Target	Primers (5`-3`)
Mouse	*Cxcl16*	Forward: CAGTGTCGCTGGAAGTTGTT<br>Reverse: TTTGGACTGCAACTGGAACC
Mouse	*Dcx*	Forward: ATTCGCAGCGGGGTGAAGCC<br>Reverse: GACTTGCTGCGGCGCATTGG
Mouse	*Il1a*	Forward: CGTGTTGCTGAAGGAGTTGC<br>Reverse: CCGACTTTGTTCTTTGGTGGC
Mouse	*Il17re*	Forward: GGGCACTGGAATGTGAAGAC<br>Reverse: CATAAGCTTCAGGCCAGCTC
Mouse	*Map2*	Forward: AAGAGAACGGGATCAACGGA<br>Reverse: CCTTGTGTTGGGCTTCCTTC
Mouse	*NeuroD1*	Forward: GCCTGCAGCTCAACCCTCGG<br>Reverse: AGCTGCGCTGTAGGCGTGTG
Mouse	*Syn1*	Forward: GGTCATTGGGCTGCAGTATG<br>Reverse: GGGTATGTTGTGCTGCTGAG
Mouse	*Gapdh*	Forward: TTTCCTCGTCCCGTAGACAAAA<br>Reverse: CGTTGAATTTGCCGTGAGTGG

Table S2 The key findings of QCM-TBI mouse model and its translational relevance to human CTE neuropathological hallmarks.

QCM-TBI Findings	Human CTE Neuropathological Hallmark	Significance / Interpretation	Key References
Force-dependent axonal ultrastructural damage	Diffuse Axonal Injury (DAI)	Microtubule disruption, axolemmal swelling, hallmark of TBI-induced axonal pathology and impaired connectivity.	Johnson et al., *Brain* 2013; Smith et al., *J Neuropathol Exp Neurol* 2003
Accumulation of Amyloid Precursor Protein (APP)	Axonal APP accumulation in injured axons	Sensitive biomarker of disrupted axonal transport; used in forensic diagnosis of TBI severity.	Gentleman et al., *Brain* 1993; Geddes et al., *Acta Neuropathol* 1999
Increased phosphorylated Tau (p-Tau)	Tauopathy characteristic of Chronic Traumatic Encephalopathy (CTE) and Alzheimer's disease	Cytoskeletal destabilization and neurofibrillary tangle formation; links repetitive or severe TBI to neurodegeneration.	McKee et al., *Brain* 2013; Johnson et al., *Nat Rev Neurol* 2012
Suppression of synaptic and neurogenesis-related gene expression	Synaptic dysfunction and impaired adult neurogenesis in TBI	Molecular basis for cognitive deficits and impaired repair mechanisms post-injury.	Ramlackhansingh et al., *Brain* 2011;
Reduction of neurofilament levels	Neurofilament light chain reduction/dysfunction	Marker of cytoskeletal damage; reflects axonal degeneration and neuronal loss.	Shahim et al., *Neurology* 2016; Zetterberg et al., *Neurology* 2013
Impact intensity-dependent pathological progression	Severity and frequency of impacts correlate with CTE pathological progression	Higher impact magnitude and repetitive injuries increase tauopathy, axonal damage, and cognitive decline; mirrored in QCM-TBI by graded pathological changes correlating with force.	Mez et al., *JAMA* 2017; Smith et al., *Nat Rev Neurol* 2013

Table S3 Information on the postmortem brain tissues of normal subjects (Normal) and chronic traumatic encephalopathy (CTE) patients.

Case	Sex	Age	Exposure Hx	CTE Stage	Age of Symptom Onset	Tauopathy
Normal 1	M	69	Not reported	N/A	N/A	N/A
Normal 2	F	83	Not reported	N/A	N/A	N/A
Normal 3	M	89	Not reported	N/A	N/A	N/A
Normal 4	F	73	Not reported	N/A	N/A	N/A
Normal 5	F	81	Not reported	N/A	N/A	N/A
Normal 6	F	81	Not reported	N/A	N/A	N/A
CTE 1	M	69	NFL, NCAA	CTE Stage III (No Aβ)	35	CTE
CTE 2	M	66	NFL, NCAA	CTE IV	56	CTE
CTE 3	M	82	NFL	CTE IV (Thal 4, Braak III, severe CAA)	67	CTE
CTE 4	M	79	NFL	CTE Stage IV	71	CTE
CTE 5	M	82	NFL	CTE Stage IV (Thal 3, Braak III)	63	CTE
CTE 6	M	70	NFL	CTE Stage IV (no Aβ)	67	CTE

Supplemantary Figure

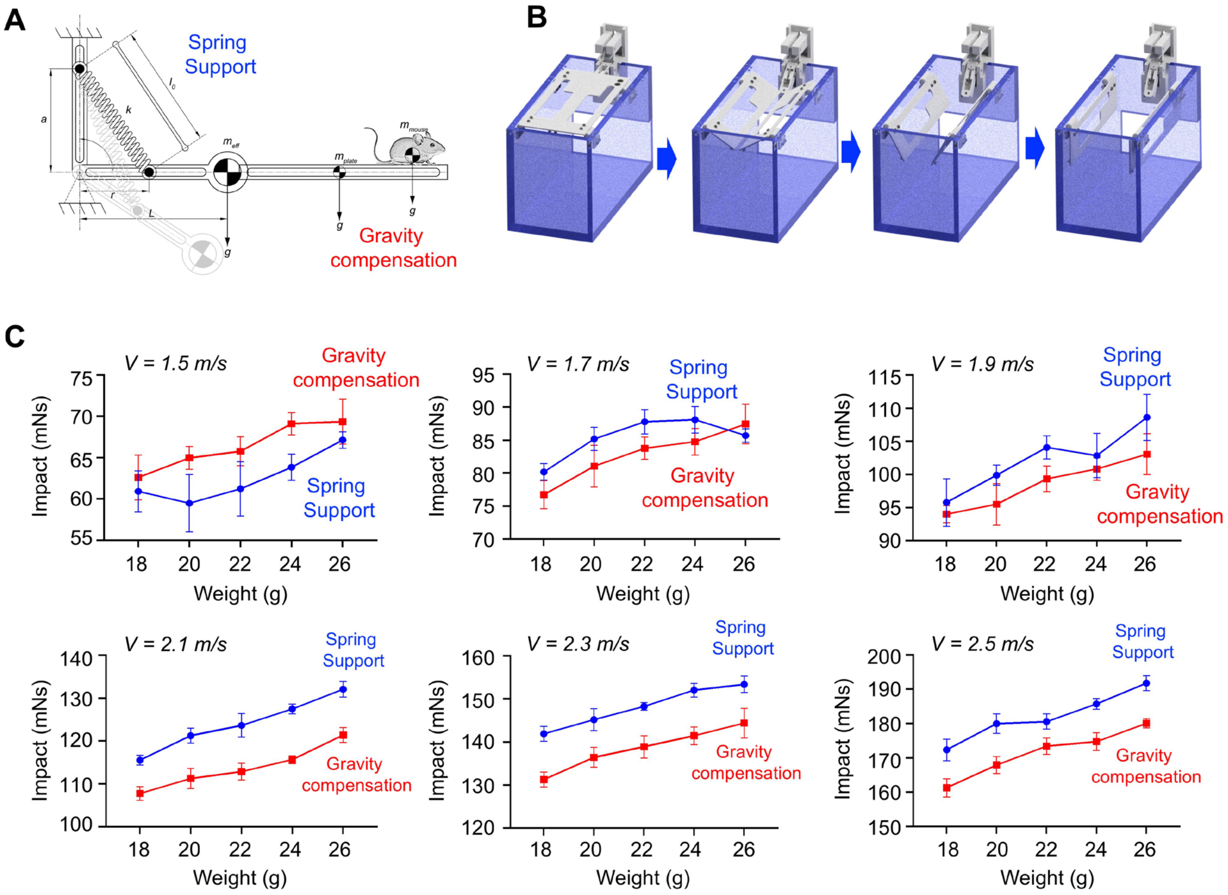

Figure S1 Design and principle of the gravity compensation mechanism. **A** Design of gravity compensation mechanism. **B** Principle of gravity compensation mechanism. **C** Comparison of the net impulse for different types of animal support (simple spring support *vs.* gravity-compensating support). The rubber mock-up models with different body weights (18, 20, 22, 24, and 26 g) were used and applied velocities varied from 1.5 m/s to 2.5 m/s. The proportional relationship between the weight and the net impulse holds only with the gravity compensation mechanism for all ranges of applying velocities, while inconsistent results were obtained with the simple spring support specifically with the low impact.

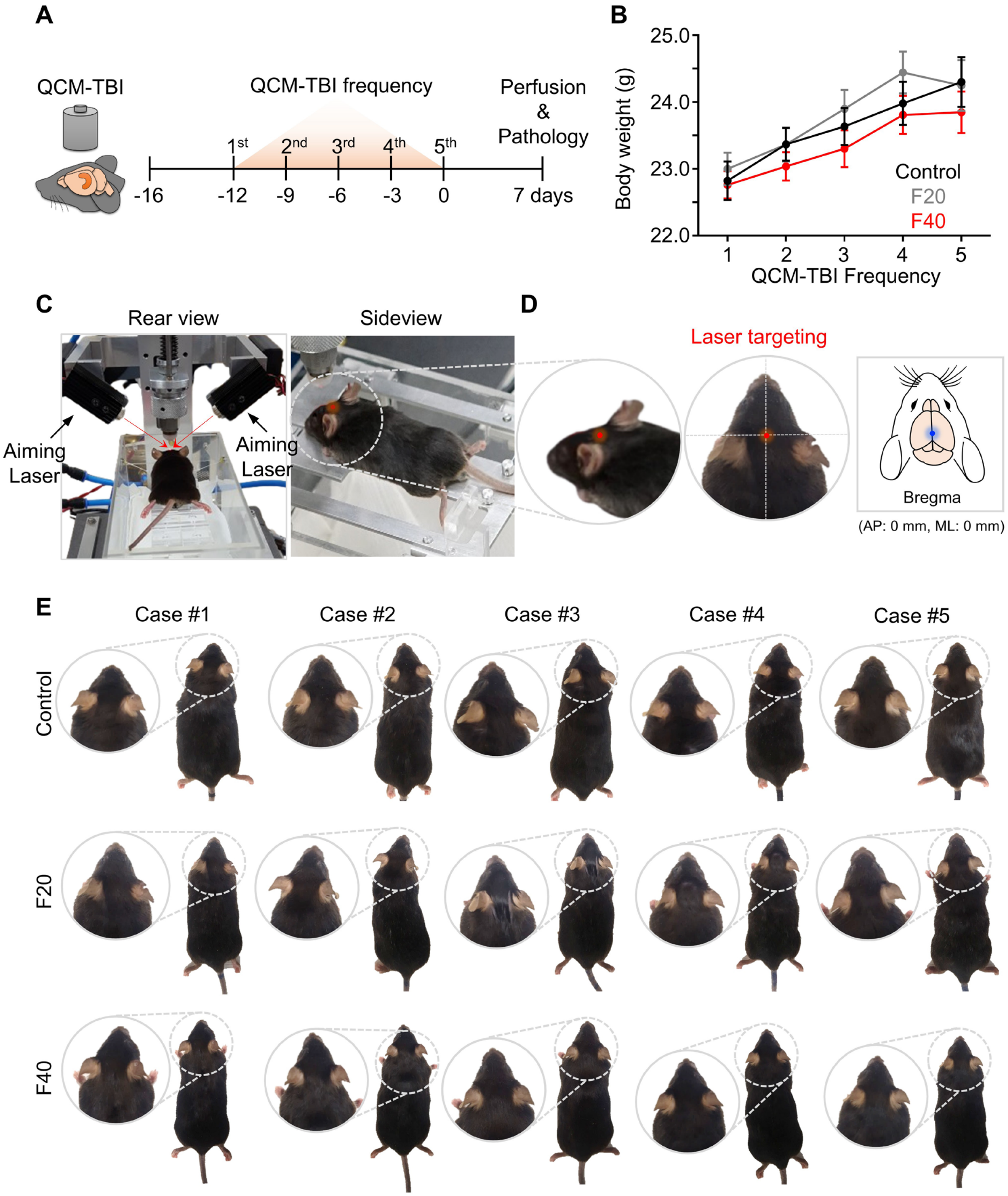

Figure S2 Overview of QCM-TBI procedure and targeting validation. **A** Schematic illustration of QCM-TBI demonstrating repeated TBIs. **B** Comparison of body weight changes during QCM-TBI procedures. **C** Detailed images illustrate a laser-guided targeting system aligned with the midline region of the dorsal skull, situated between bregma and lambda. **D** High-magnification images illustrate the laser targeting site (*red*) on the head. The schematic diagram illustrates the precise location of the targeting site of QCM-TBI (Anterior-Posterior: 0 mm; Medial-Lateral: 0 mm; *blue*). **E** Detailed images depict the skin contusion on the head resulting from QCM-TBI.

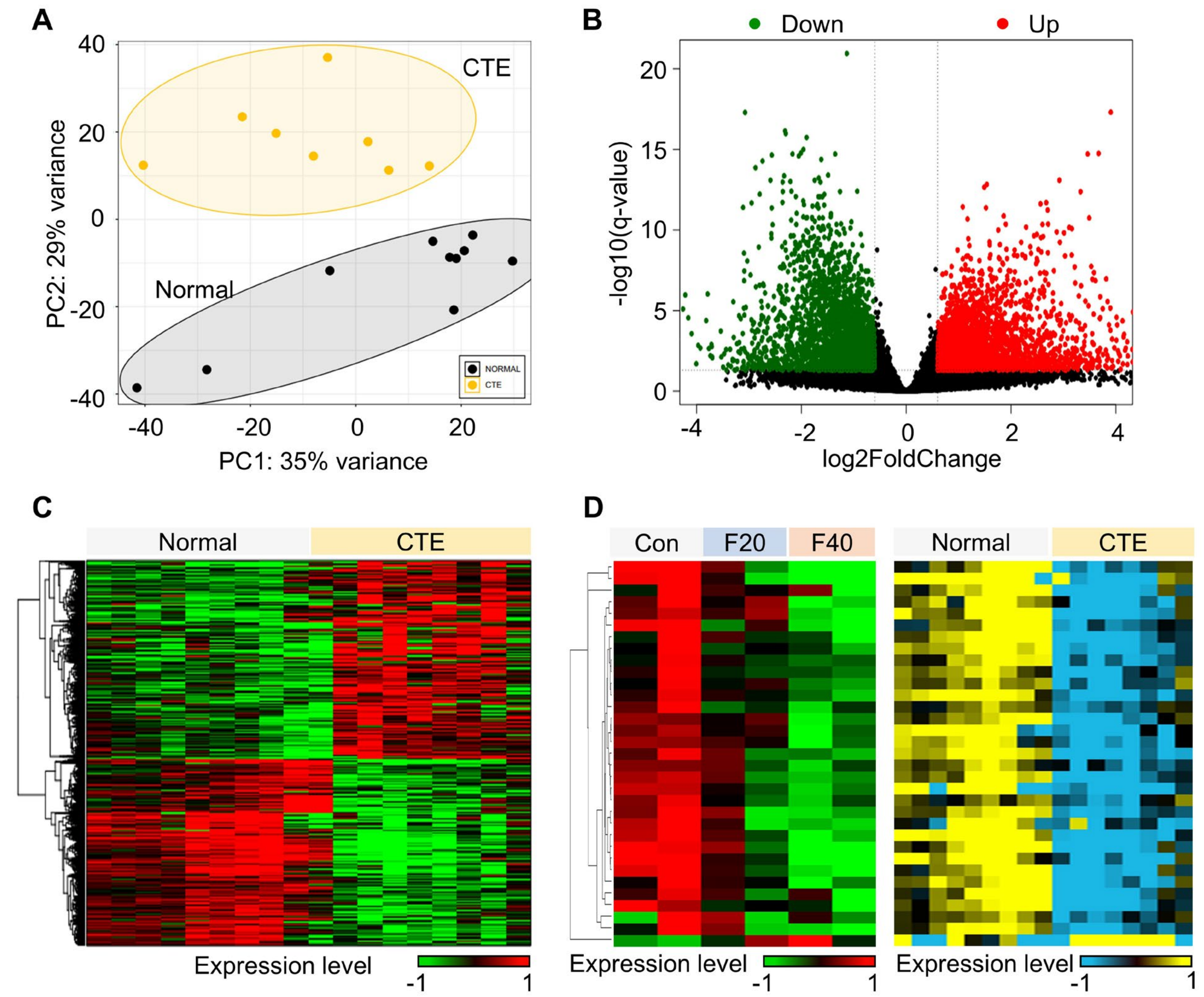

Figure S3 Gene expression profiling of CTE. **A** Principal component analysis of 10 normal and 8 CTE samples. **B** Volcano plot of differentially expressed genes (DEGs) in CTE. Red dot indicates the up-regulated genes in CTE and green dot represents the down-regulated genes in CTE compared to normal samples. **C** Heatmap of gene expression comparison between the normal and CTE samples. **D** Expression levels of 33 common neuronal genes in both QCM-TBI and CTE.

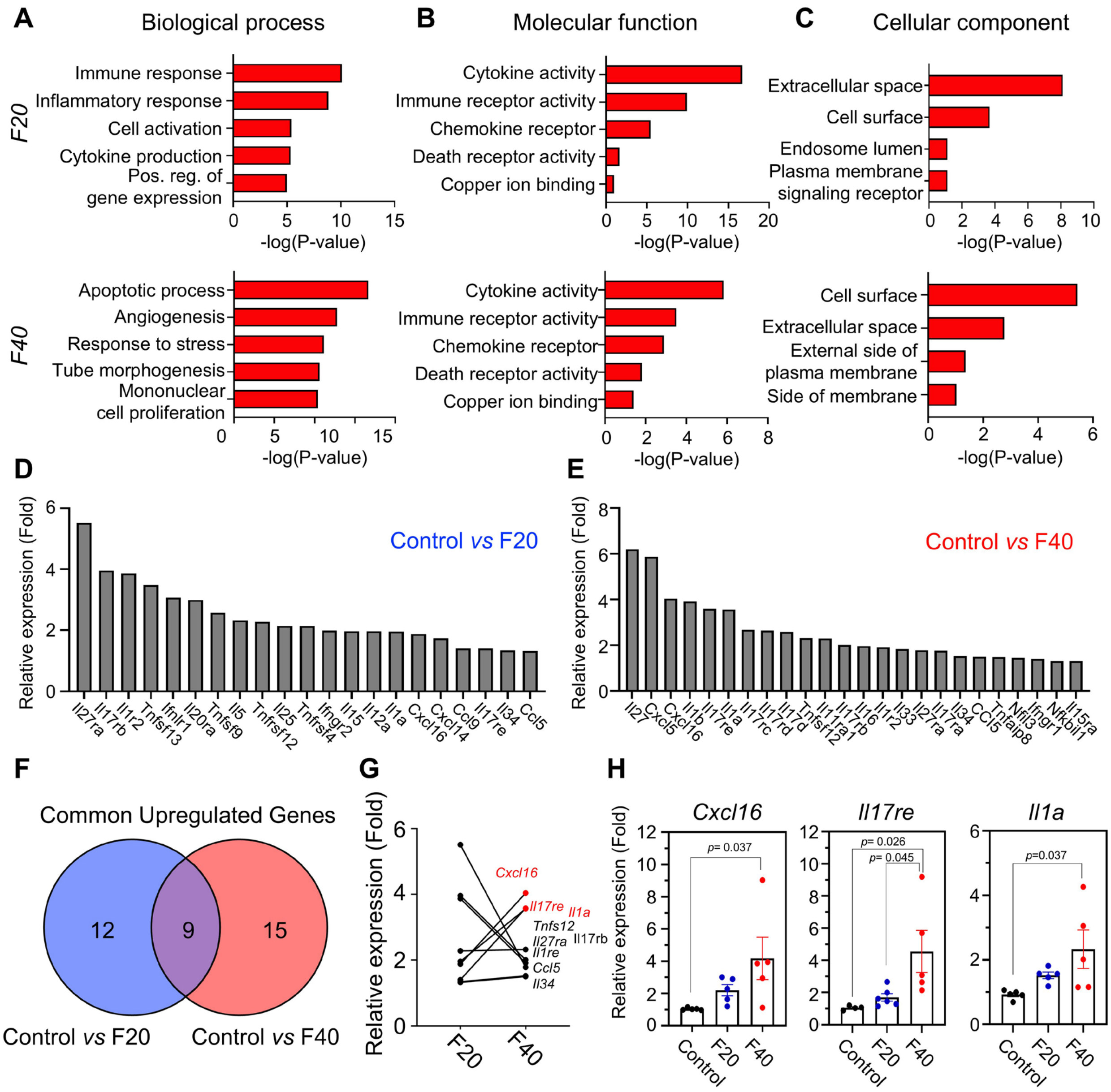

Figure S4 Gene expression profiling is associated with inflammation in QCM-TBI. **A-C** GO enrichment analysis of upregulated genes in the hippocampus of F20 (*upper panel*) and F40 (*lower panel*) group mice relative to control. Bar plot represents significantly enriched biological process (BP; A), molecular function (MF; B) and cellular component (CC; C) terms ranked by –log(P-value). GO terms enriched among upregulated genes, including pathways related to immune response, inflammatory response, cytokine activity. Only GO terms with adjusted p-value < 0.05 are shown. **D, E** Bar charts illustrate the relative expression levels and DEGs in F20 (D) and F40 (E) compared to control. **F** A Venn diagram demonstrates the number of overlapping genes among upregulated DEGs in QCM-TBI at F20 (*blue*) and F40 (*red*) compared with control, with 9 genes commonly upregulated (*purple*). **G** A graph depicting relative expression indicates that *Cxcl16*, *Il17re*, and *Il1a* are upregulated in a force-dependent manner. **H** qRT-PCR analysis confirms an upregulation in the expression levels of *Cxcl16*, *Il17re*, and *Il1a* in the dorsal hippocampus of the QCM-TBI mouse model (control, n = 5 mice; F20, n = 5 mice; F40, n = 5 mice). Data are presented as mean ± SEM.

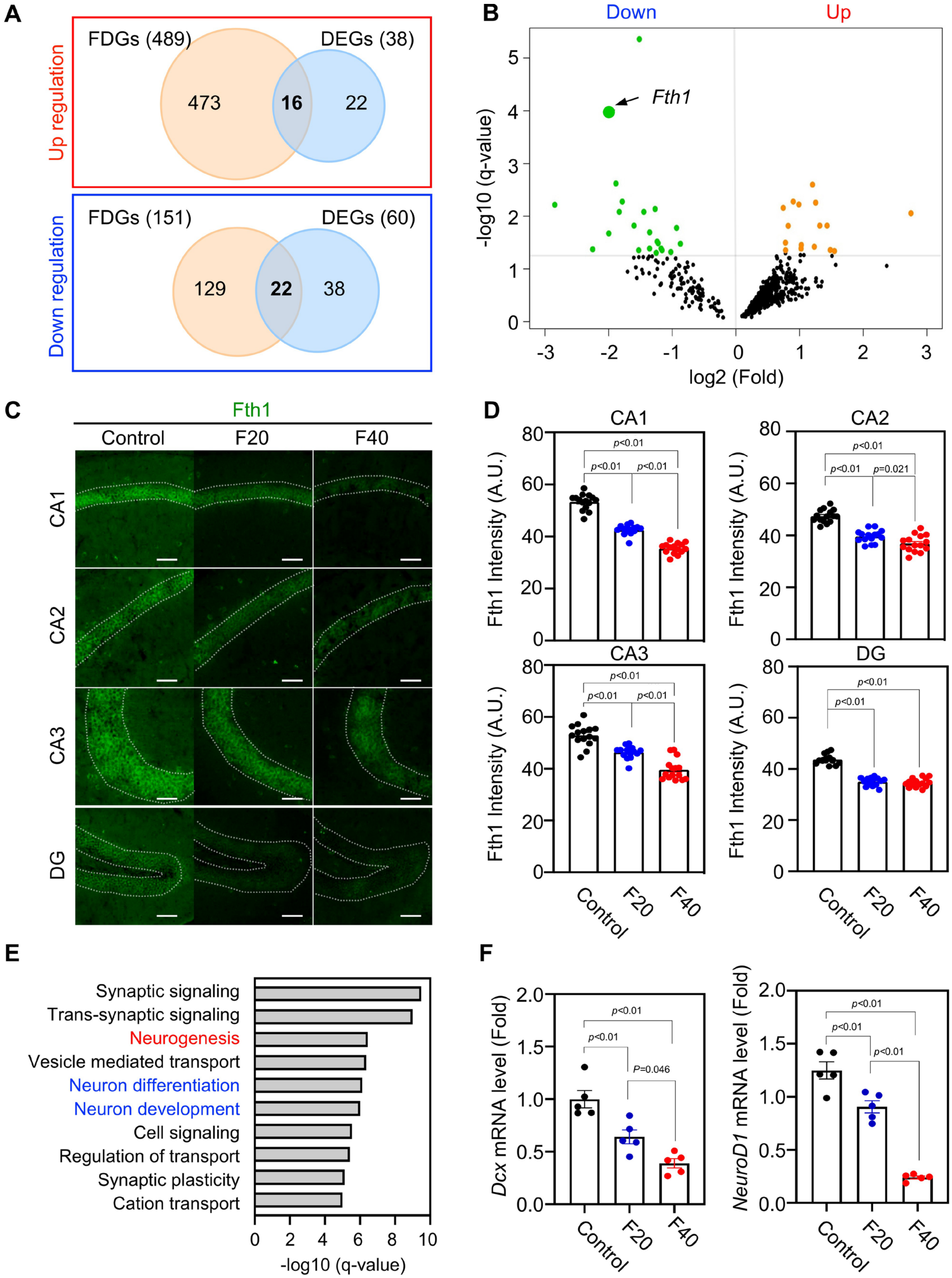

A
Up regulation
FDGs (489)
DEGs (38)
473
16
22
Down regulation
FDGs (151)
DEGs (60)
129
22
38
B
Down
Up
Fth1
-log10 (q-value)
log2 (Fold)
C
Fth1
Control
F20
F40
CA1
CA2
CA3
DG
D
CA1
CA2
CA3
DG
Fth1 Intensity (A.U.)
p<0.01
p=0.021
Control
F20
F40
E
Synaptic signaling
Trans-synaptic signaling
Neurogenesis
Vesicle mediated transport
Neuron differentiation
Neuron development
Cell signaling
Regulation of transport
Synaptic plasticity
Cation transport
-log10 (q-value)
F
Dcx mRNA level (Fold)
NeuroD1 mRNA level (Fold)
p<0.01
P=0.046
Control
F20
F40

Figure S5 Gene expression profiling of QCM-TBI. **A** Venn diagram indicates the number of overlapped genes between force-dependent genes and differentially expressed genes in QCM-TBI (F20 and F40) compared to control. **B** Volcano plot of up- and down-regulated genes in QCM-TBI. Yellow-green dots indicate down-regulated genes and orange dots represent up-regulated genes in QCM-TBI. Fth1 represented by green dots. **C** Immunoreactivity of Ferritin heavy chain 1 (Fth1) was significantly decreased in hippocampus of QCM-TBI mice compared to control. Scale bar: 50 μm. **D** Quantitation data of Fth1 intensity of hippocampus in QCM-TBI and control mice (lower panel). **E** Expression levels of 33 common neuronal genes in both QCM-TBI and CTE. **F** Quantitative RT-PCR analysis verified reduction of *Dcx* and *NeuroD1* expression levels in the hippocampus of the QCM-TBI mouse model (control, $n = 5$; F20, $n = 5$; F40, $n = 5$). Data represent the mean ± SEM.

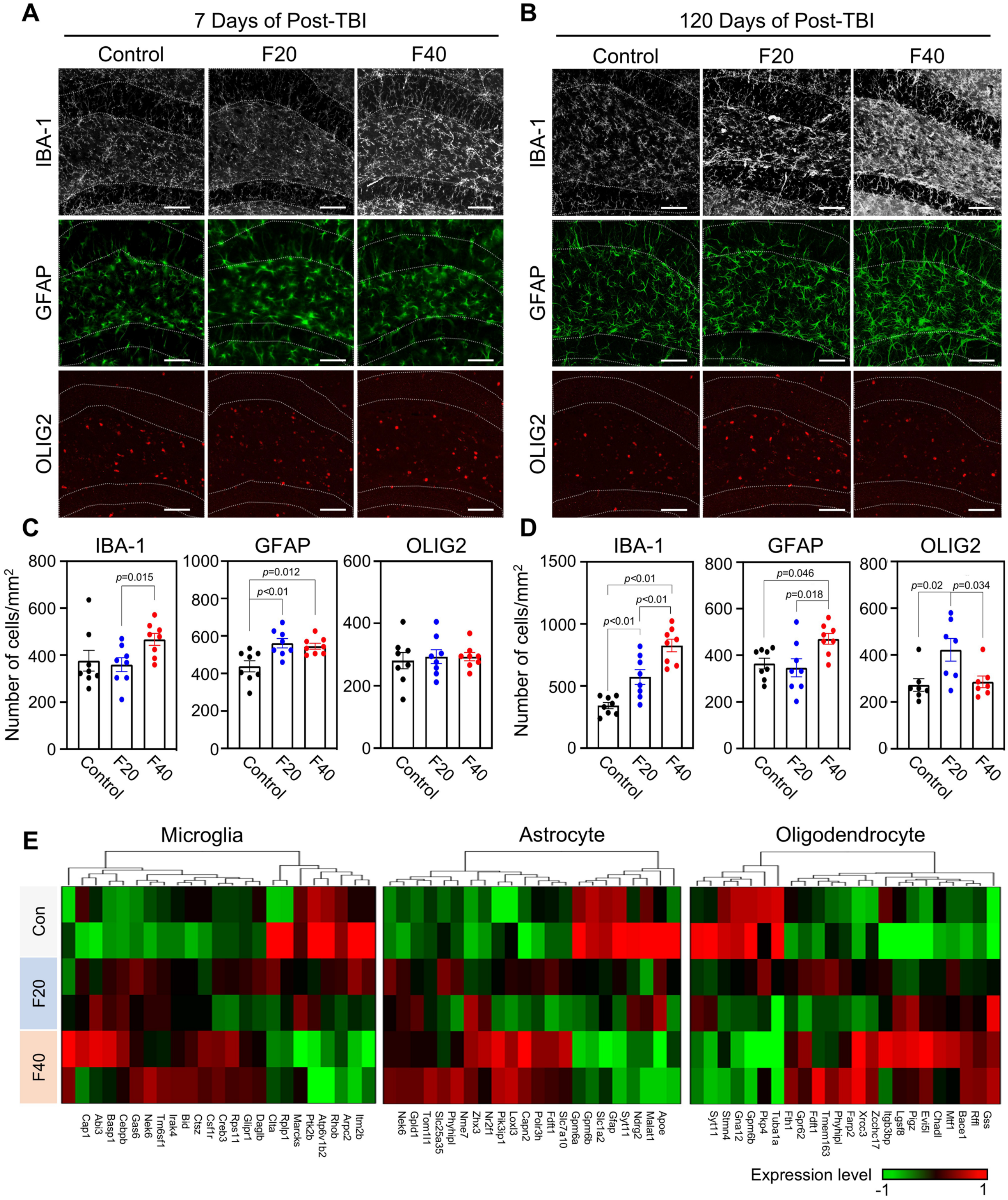

Figure S6 Astrocyte-, oligodendrocyte-, microglia marker- positive cells of QCM-TBI. **A, C** Immunohistochemical staining images (A) and quantitation (C) of number of IBA-1-, GFAP- and OLIG2-positive cells in DG of at 7days after QCM-TBI and control mice (control, *n* = 5; F20, *n* = 5; F40, *n* = 5).

Scale bars: 50 μm. **B, D** Immunohistochemical staining images (B) and quantitation (D) of IBA-1, GFAP and OLIG2-positive cells at 120days after QCM-TBI and control mice. Scale bars: 50 μm. **E** Identification of cell-type specific genes in QCM-TBI. Heatmap illustrates the expression levels of cell-type specific FDGs (40 neurons, 22 astrocytes, 23 oligodendrocytes and 23 microglia) in QCM-TBI. Data represent the mean ± SEM.

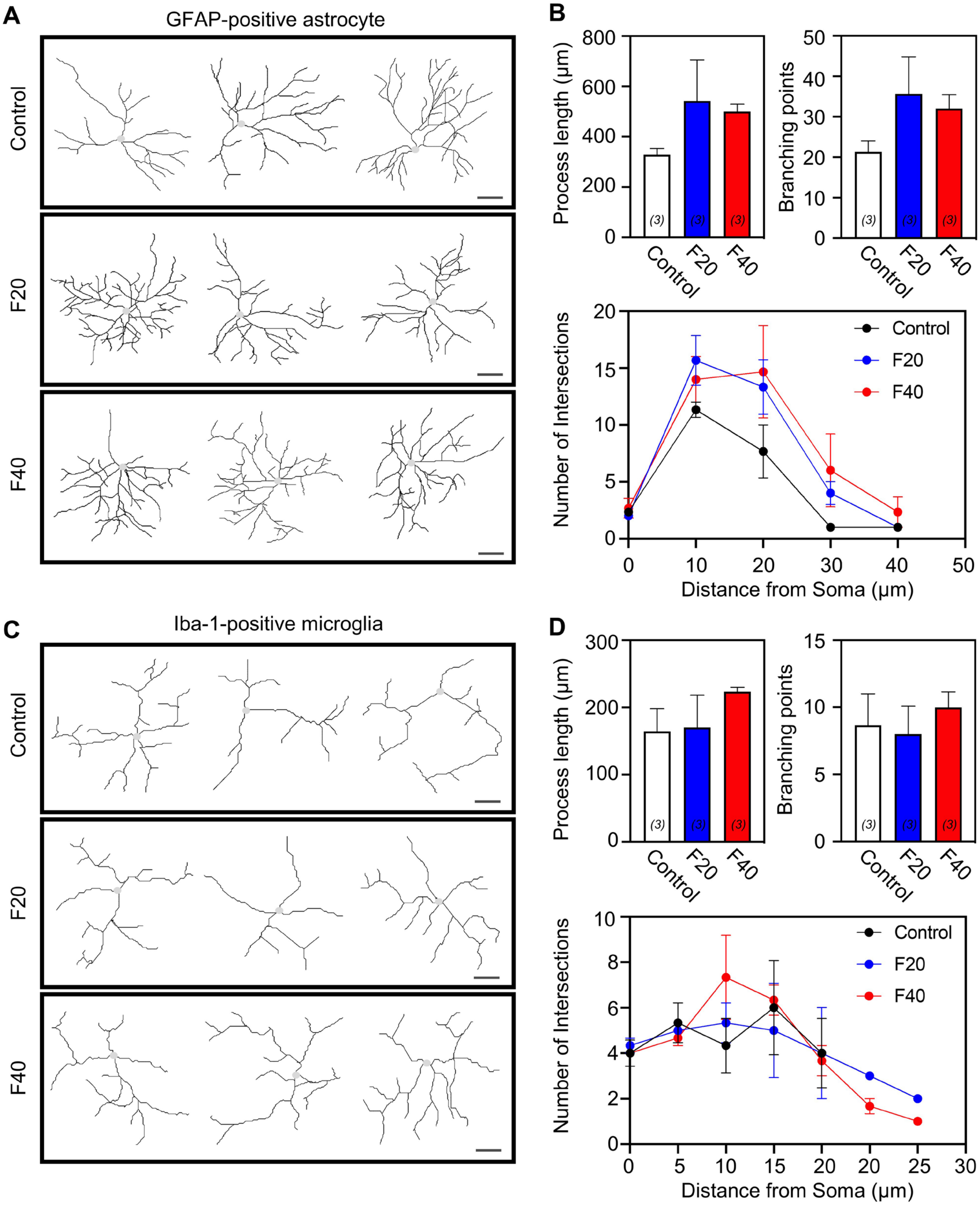

Figure S7 Process of GFAP and IBA-1-positive cells in QCM-TBI mice. **A** 3D-reconstruction image of GFAP-positive cells in DG of QCM-TBI and control mice. Scale bars: 10 μm. **B** Quantitation of the process length and number of branching points and intersections in GFAP-positive cells in DG of QCM-TBI and

control mice. **C** 3D-reconstruction images of IBA-1-positive cells in DG of QCM-TBI and control mice. Scale bars: 10 μm. **D** Quantitation of the process length and number branching points and intersections of IBA-1-positive cells in DG of QCM-TBI and control mice. Data represent the mean ± SEM.

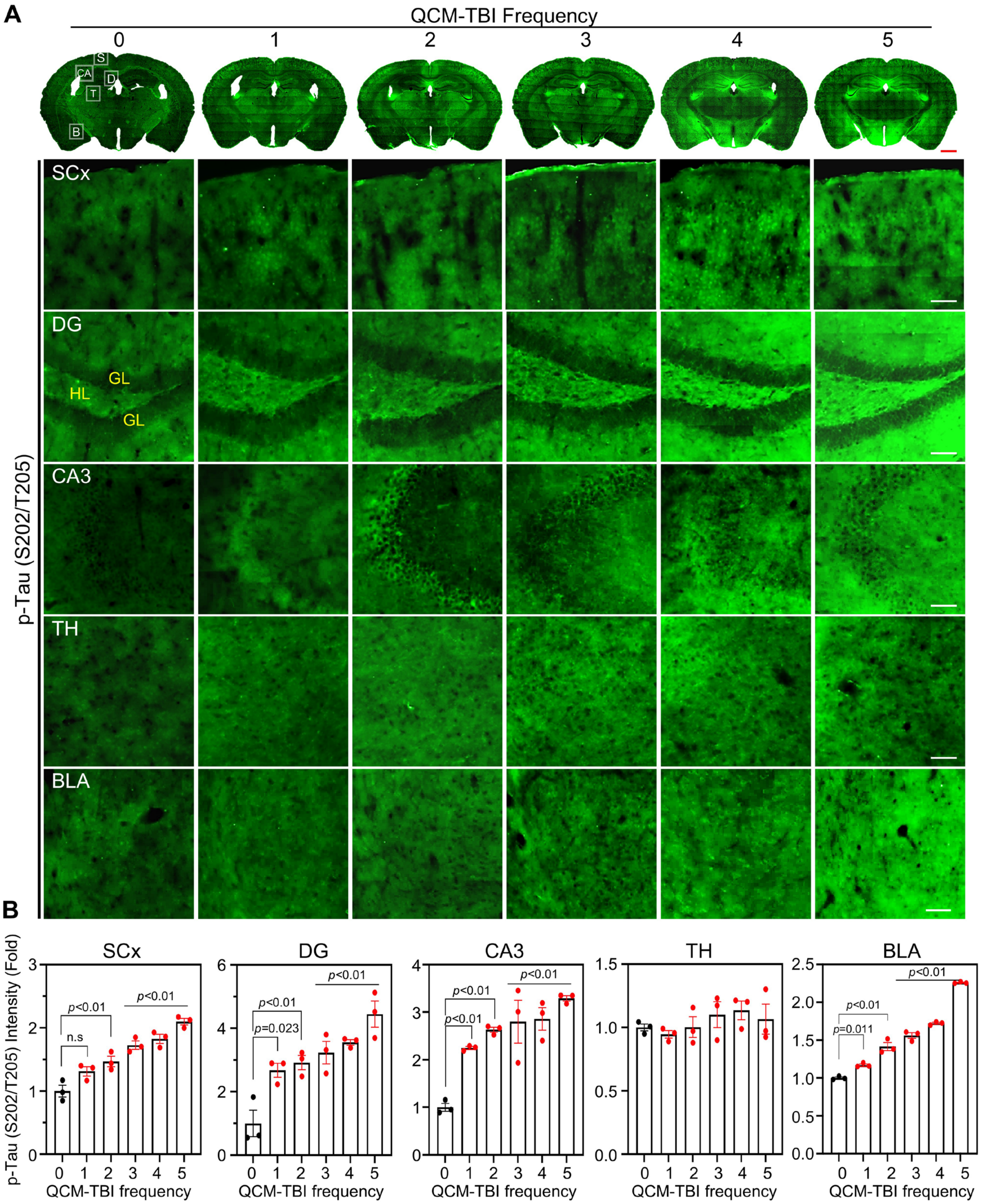

Figure S8 QCM-TBI influences p-Tau (S202/T205) levels in the mouse brain in a manner dependent on the frequency of impacts. **A, B** QCM-TBI increased p-Tau (S202/T205) levels in the SCx, DG, CA3, TH, and BLA but not in the TH of the mouse brain. Scale bars: 1 mm (*red*); 50 µm (*black*). Data are presented as mean ± SEM.

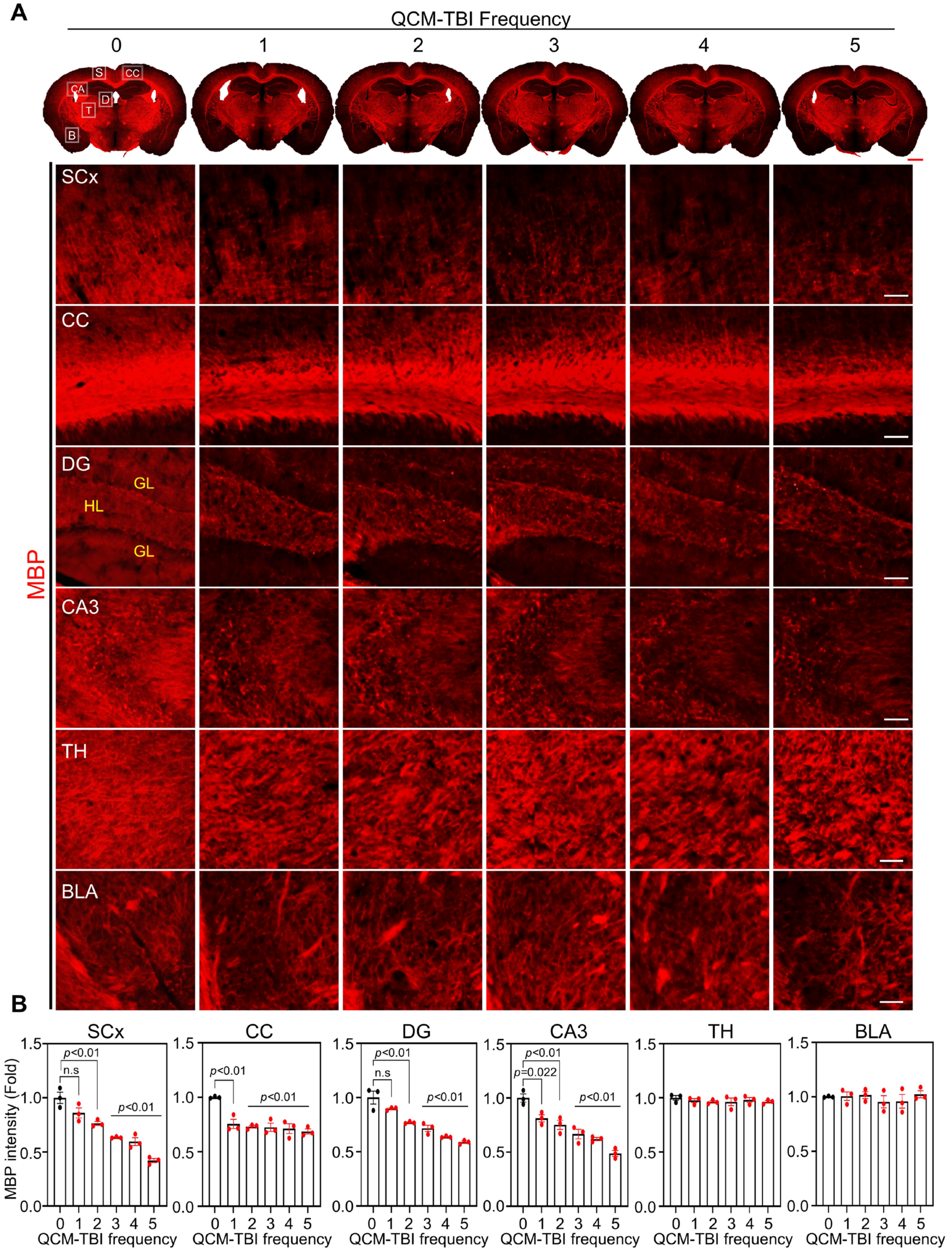

Figure S9 QCM-TBI influences myelin basic protein (MBP) levels in the mouse brain in a manner dependent on the frequency of impacts. **A, B** QCM-TBI decreased MBP levels in the SCx, CC, DG and

CA3 but not in the TH and BLA of the mouse brain. Scale bars: 1 mm (*red*); 50 µm (*black*). Data are presented as mean ± SEM.

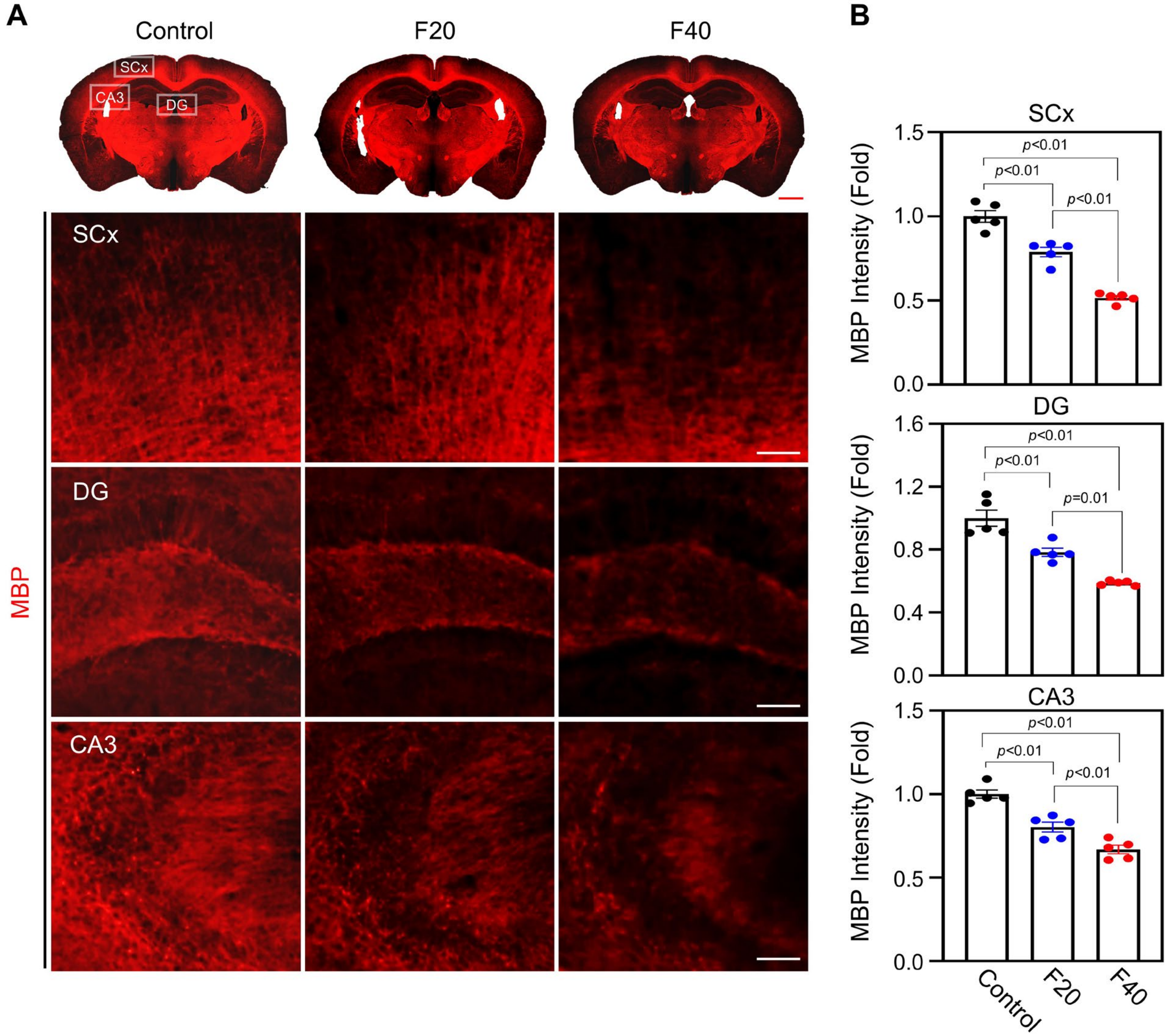

Figure S10 QCM-TBI influences MBP levels in the mouse brain of QCM-TBI. **A, B** QCM-TBI decreased MBP levels in the SCx, DG and CA3 of the brain in QCM-TBI mice. Scale bars: 1 mm (*red*); 50 µm (*white*). Data are presented as mean ± SEM.

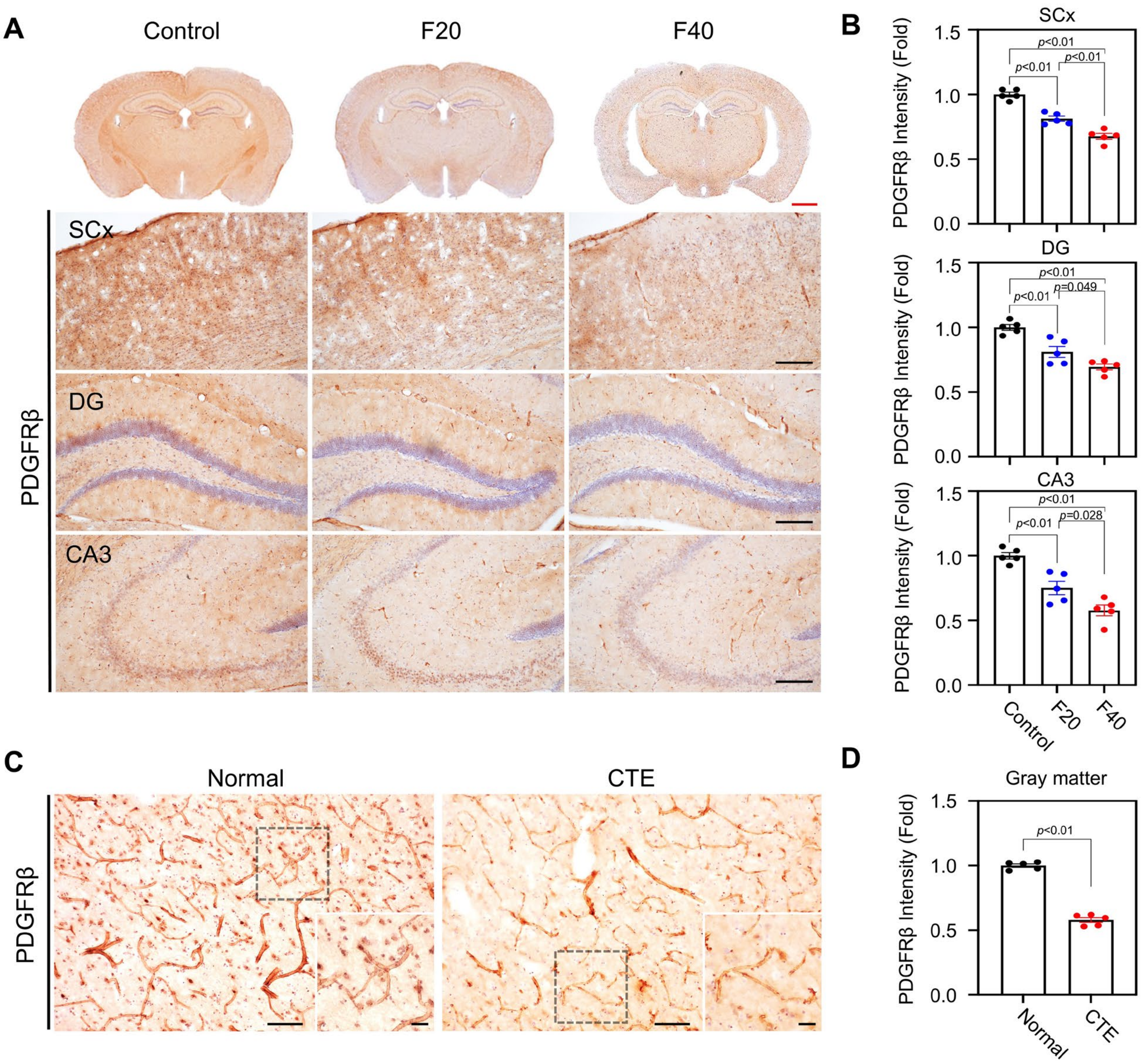

Figure S11 QCM-TBI alters PDGFRβ expression in blood vessels in the mouse brain. **A** Immunoreactivity of PDGFRβ decreased by QCM-TBI in the SCx, DG and CA3 of QCM-TBI and control mice. **B** The densitometry analysis showed that PDGFRβ was significantly decreased by QCM-TBI (Control, $n = 5$ mice; F20, $n = 5$ mice; F40, $n = 5$ mice). Scale bars: 1 mm (*red*); 50 µm (*black*). **C** PDGFRβ expression in blood vessels was reduced in the cortical regions of CTE patients and normal. **D** The densitometry analysis showed that PDGFRβ was significantly decreased in gray matter of cortical regions of CTE compared with normal (normal, $n = 5$; CTE, $n = 5$). Scale bar: 50 µm (*outlet*); 20 µm (*inlet*). Data are presented as mean ± SEM.

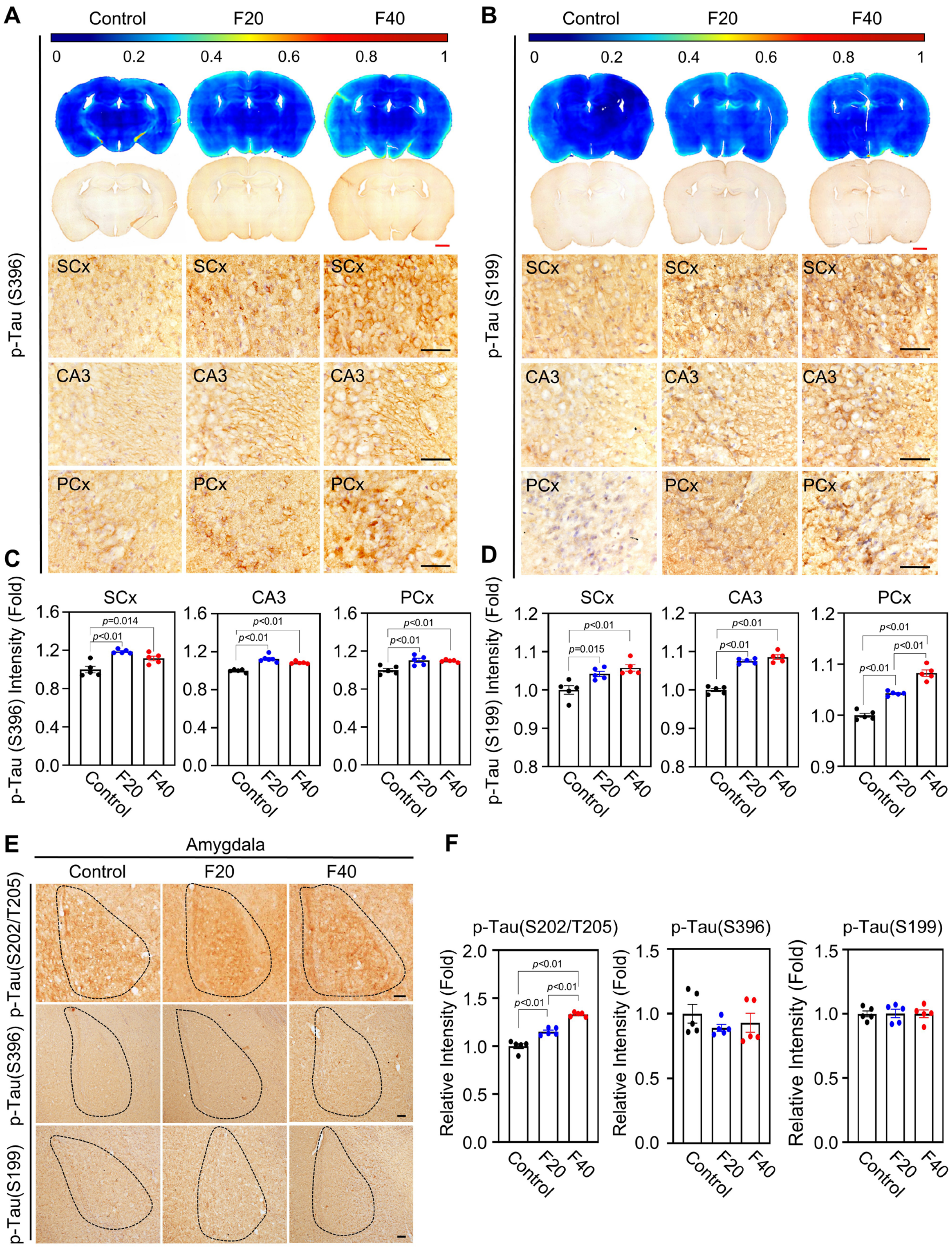
A
Control
F20
F40
0
0.2
0.4
0.6
0.8
1
p-Tau (S396)
SCx
CA3
PCx
B
p-Tau (S199)
C
p-Tau (S396) Intensity (Fold)
p=0.014
p<0.01
Control
F20
F40
D
p-Tau (S199) Intensity (Fold)
p=0.015
E
Amygdala
p-Tau(S202/T205)
p-Tau(S396)
p-Tau(S199)
F
Relative Intensity (Fold)

Figure S12 QCM-TBI alters p-Tau (S396 and S199) levels in the mouse brain. **A, C** QCM-TBI increased p-Tau (S396) levels in the SCx, CA3 and PCx in the mouse brain. Scale bars: 1 mm (*red*), 50 μm (*black*). The bar graphs show that p-Tau (S396) was significantly elevated in the QCM-TBI-induced mouse brain. **B** QCM-TBI increased p-Tau (S199) levels in the SCx, CA3 and PCx of the mouse brain. **D** The bar graphs show that p-Tau (S199) was significantly elevated in the SCx, CA3 and PCx of the QCM-TBI-induced mouse brain in an impact force-dependent manner. Scale bars: 1 mm (*red*), 50 μm (*black*). **E, F** p-Tau (S202/T205) increased in the basolateral amygdala (BLA) of QCM-TBI mice, but p-Tau (S396 and S199) were not changed. Scale bar: 100 μm. Data represents the mean ± SEM.

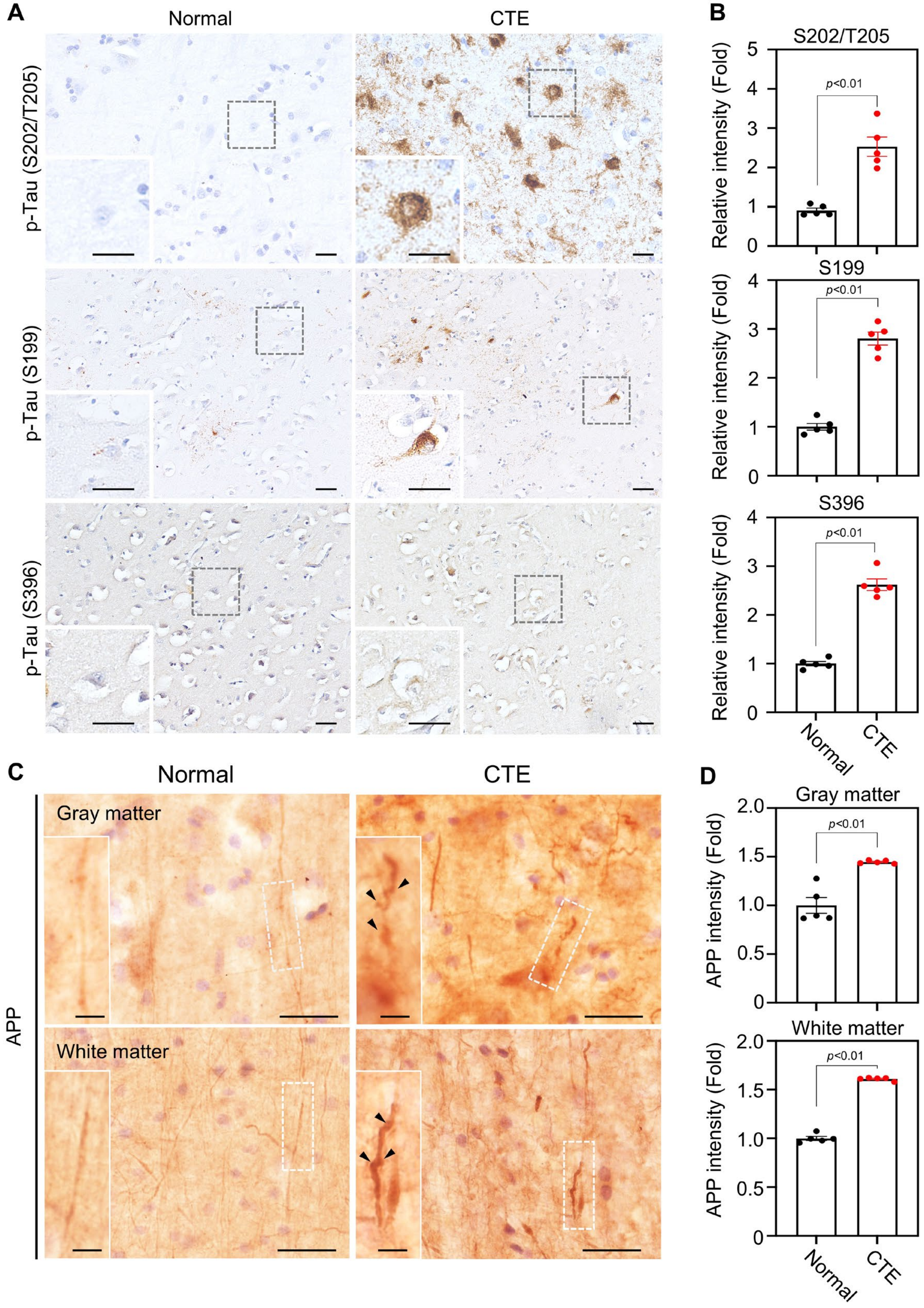
A
Normal
CTE
p-Tau (S202/T205)
p-Tau (S199)
p-Tau (S396)
B
S202/T205
S199
S396
Relative intensity (Fold)
p<0.01
Normal
CTE
C
Normal
CTE
APP
Gray matter
White matter
D
Gray matter
White matter
APP intensity (Fold)
p<0.01
Normal
CTE

Figure S13 p-Tau (S202/T205), p-Tau (S199) and p-Tau (S396) levels increased in the cortex of CTE patients. **A** p-Tau (S202/T205, S199 and S396) levels in the cortex of CTE patient and normal. Scale bars: 20 μm (*outlet*), 20 μm (*inlet*). **B** The bar graphs show that p-Tau (S202/T205, S199 and S396) were significantly elevated in the cortex of CTE patients compared with normal. **C** Immunoreactivity of APP was increased in the cortex of CTE patients compared with normal. 50 μm (*outlet*), 20 μm (*inlet*). **D** The densitometry analysis showed that APP was significantly increased in gray matter and white matter in the cortex of CTE patients compared with normal. Data are presented as mean ± SEM.

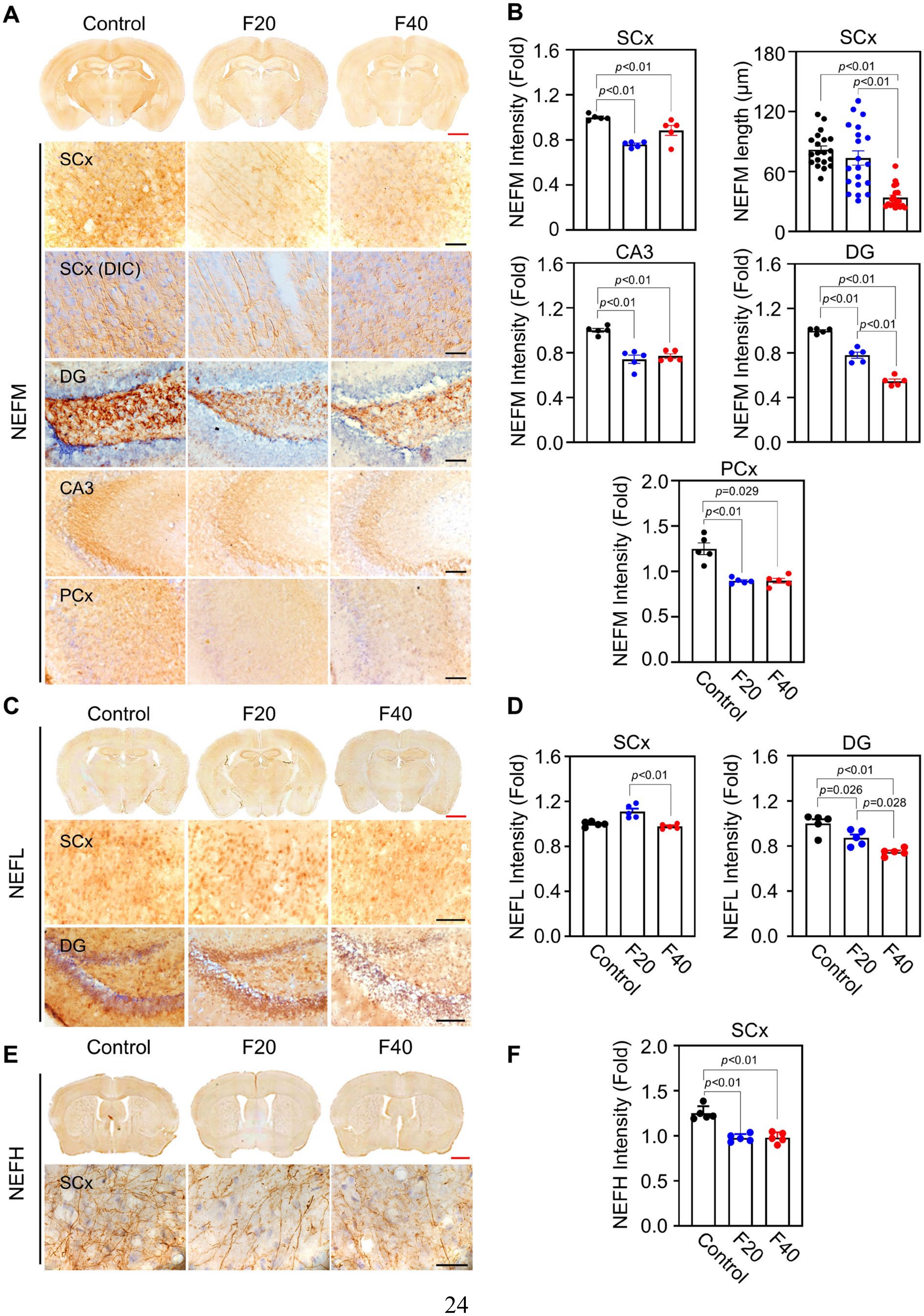
A
Control
F20
F40
SCx
SCx (DIC)
DG
CA3
PCx
NEFM
B
SCx
NEFM Intensity (Fold)
p<0.01
p<0.01
NEFM length (μm)
p<0.01
p<0.01
CA3
p<0.01
p<0.01
DG
p<0.01
p<0.01
p<0.01
PCx
p=0.029
p<0.01
Control
F20
F40
C
NEFL
D
NEFL Intensity (Fold)
p<0.01
p<0.01
p=0.026
p=0.028
E
NEFH
F
NEFH Intensity (Fold)
p<0.01
p<0.01

Figure S14 QCM-TBI alters neurofilament medium (NEFM), heavy (NEFH) and light polypeptide (NEFL) levels in the mouse brain. **A, B** Immunoreactivity of NEFM decreased by QCM-TBI in the SCx, DG, CA3 and PCx of the mouse brain. The densitometry analysis showed that NEFM was significantly decreased by QCM-TBI. Scale bars: 1 mm (*red*); 50 µm (*black*). **C, D** Immunoreactivity of NEFL was decreased by QCM-TBI in the DG but not in the SCx of the mouse brain. The densitometry analysis showed that NEFL was significantly decreased by QCM-TBI in the DG but not in the SCx. Scale bars: 1 mm (*red*); 50 µm (*black*). **E, F** Immunoreactivity of NEFH was decreased by QCM-TBI in the SCx of the mouse brain. The densitometry analysis showed that NEFH was significantly decreased by QCM-TBI in the SCx. Scale bars: 1 mm (*red*); 50 µm (*black*). A total of 50 cells (10 cells/case) were examined from $n = 5$. Data represent the mean ± SEM

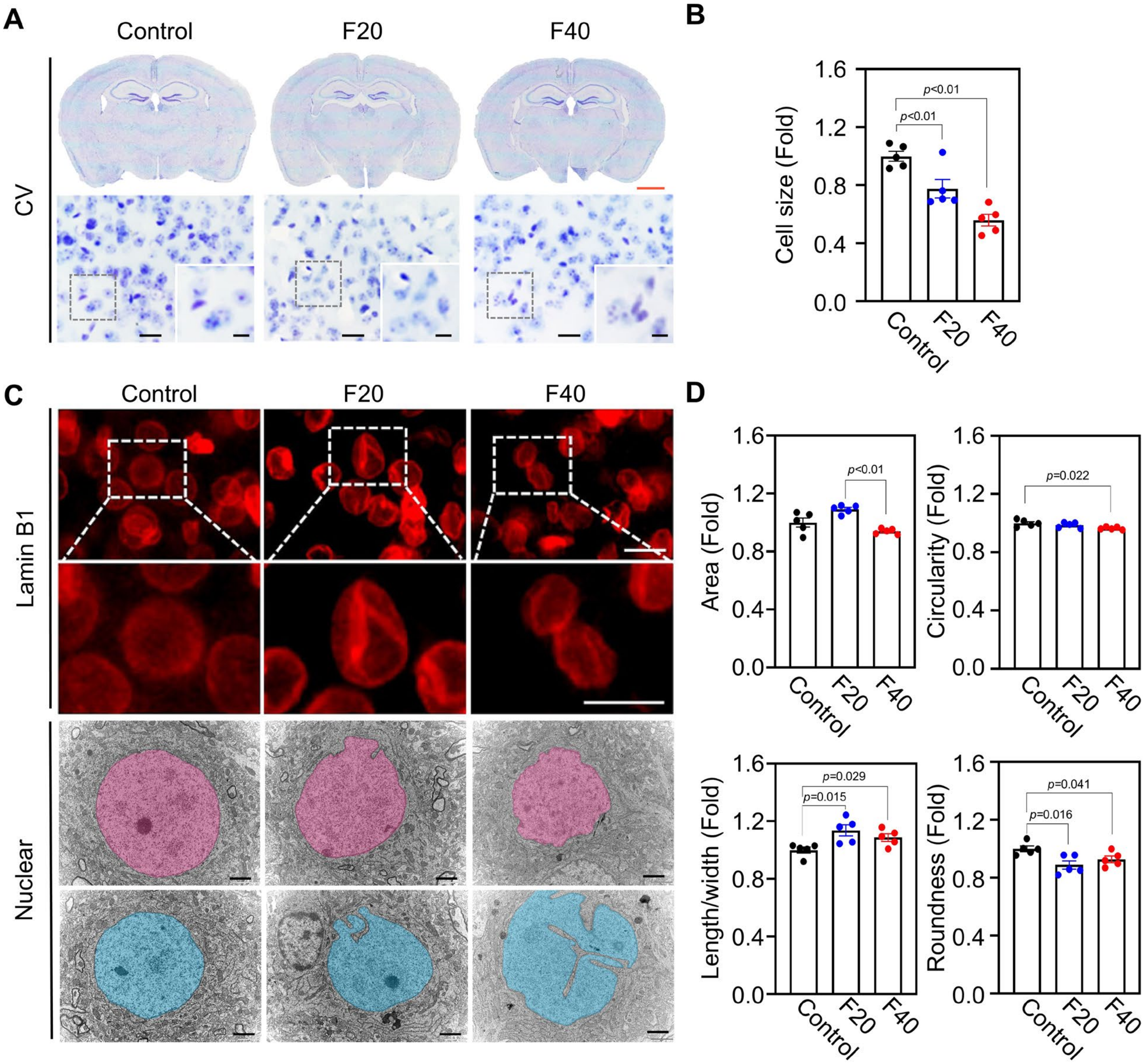

Figure S15 QCM-TBI alters soma size and ultrastructure of nuclear pathology. **A** Relative cell sizes were significantly reduced in QCM-TBI mice compared with control mice. Significantly different from the control. Scale bars: 1 mm (*red*); 20 µm (*outlet, black*); 20 µm (*inlet, black*). **B** Bar graph shows the relative cell size changes in QCM-TBI-induced mice. A total of 50 cells (10 cells/case) were examined from $n = 5$. **C** Immunoreactivity of Lamin B1 showed structural changes of the nuclear envelope. Scale bar: 20 µm. The ultrastructure of nucleus was altered by QCM-TBI. Representative images showing invagination of a single nucleus in the QCM-TBI-induced mouse models (F20 and F40). More invaginated nuclei were observed in F40 than in F20 and the control. Scale bar: 2 µm. **D** Roundness and circularity of the nuclei were decreased by QCM-TBI. A total of 50 cells (10 cells per mouse) were examined from $n = 5$ mice. Data represent the mean ± SEM.

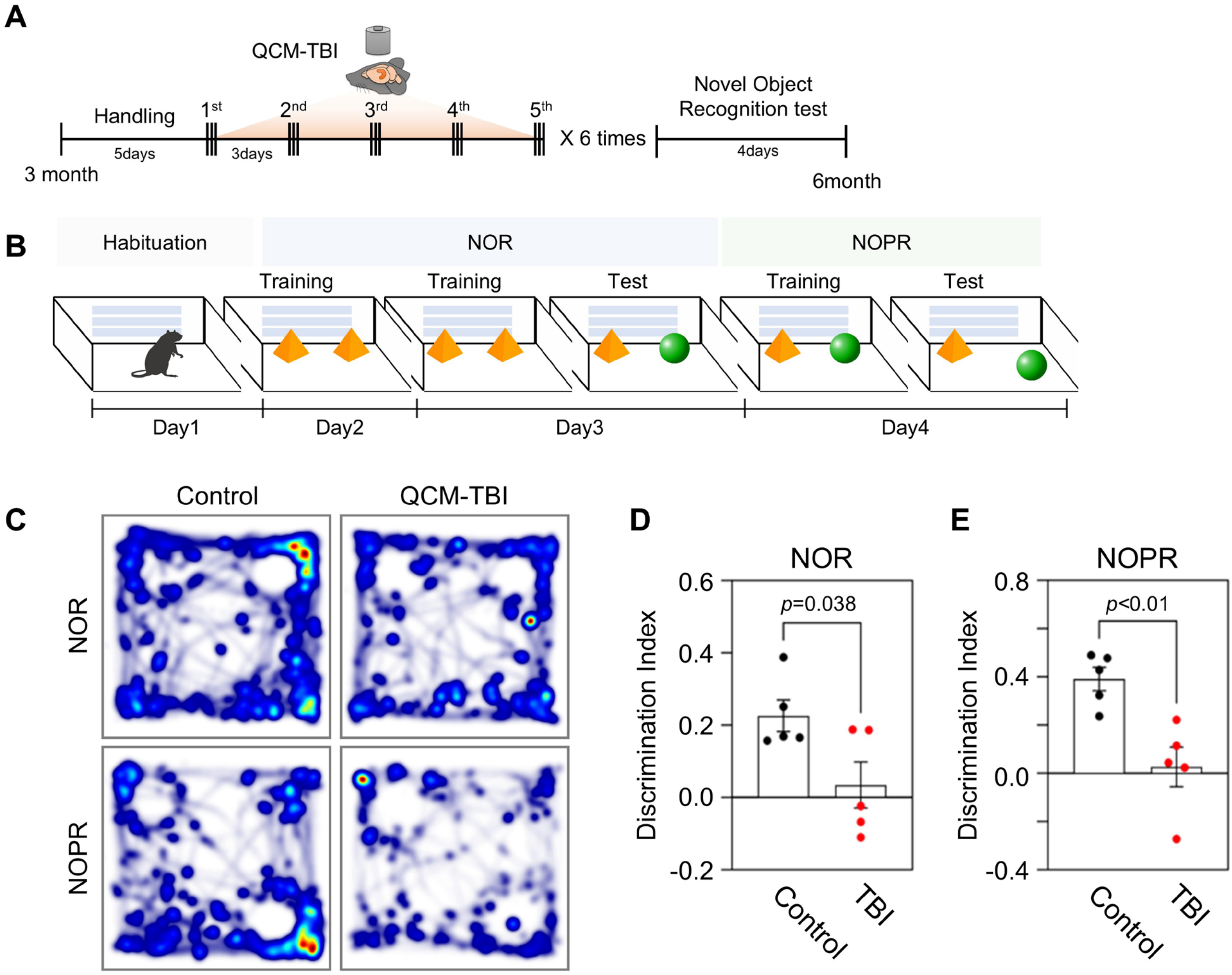

Figure S16 Cognitive functions are impaired in QCM-TBI mice. **A** Experimental scheme of the modified QCM-TBI model for measuring cognitive function by novel object recognition (NOR) and novel object place recognition (NOPR) tests. **B** Schematic image of NOR and NOPR tests. **C** Representative heatmap of movement in the NOR and NOPR tests. Red indicates increased time spent, and blue indicates minimal time spent during the trials. **D, E** Average discrimination index for NOR (D) and NOPR (E) tests of QCM-TBI mice (n = 5 per group). This data shows a decline in memory in QCM-TBI mice compared to the control. Data are presented as mean ± SEM.
